# Targeting Microglial CD49a Inhibits Neuroinflammation and Demonstrates Therapeutic Potential for Parkinson's Disease

**DOI:** 10.1002/advs.202515138

**Published:** 2025-12-29

**Authors:** Huanpeng Lu, Yunmin Zhu, Xi Wang, Zelin Wu, Zijian Xu, Rongqing Chen, Yanwu Guo

**Affiliations:** ^1^ Neurosurgery Center, Department of Functional Neurosurgery, The National Key Clinical Specialty, Guangdong Provincial Key Laboratory on Brain Function Repair and Regeneration, Zhujiang Hospital Institute for Brain Science and Intelligence, Zhujiang Hospital Southern Medical University Guangzhou China; ^2^ General Hospital of Southern Theater Command of PLA, The First School of Clinical Medicine Southern Medical University Guangzhou China; ^3^ Department of Neurology, Nanfang Hospital Southern Medical University Guangzhou China; ^4^ Neurosurgery Center, The National Key Clinical Specialty, Guangdong Provincial Key Laboratory on Brain Function Repair and Regeneration, Zhujiang Hospital Institute for Brain Science and Intelligence, Zhujiang Hospital Southern Medical University Guangzhou China

**Keywords:** CD49a, integrin, microglia, neuroinflammation, Parkinson's disease, peptide

## Abstract

Persistent microglial activation drives chronic neuroinflammation, a characteristic pathological hallmark of neurodegenerative disorders, including Parkinson's disease (PD). Although integrin receptor CD49a (*Itga1* gene) serves as a canonical biomarker of tissue‐resident immune populations, its microglial expression patterns, functions, and signaling pathways have not been elucidated. In this study, we aim to investigate the impact of CD49a in hyperactivated microglia on PD pathogenesis and elucidate downstream signaling pathways. Specifically, we demonstrate microglia‐enriched CD49a expression with pathologically significant upregulation particularly in microglia adopting chronically activated states. Specific *Itga1* knockdown attenuates microglial hyperreactivity and markedly improves motor deficits in PD mouse models. Mechanistically, transcriptomic profiling of isolated microglia from mouse substantia nigra reveals significant enrichment in neurodegeneration and inflammation pathways, with PGAM5 emerging as a central regulatory node. Conditional microglial *Itga1* knockdown ameliorates mitochondrial dysfunction and suppresses NLRP3 inflammasome assembly via PGAM5 downregulation, thereby preserving dopaminergic neurons from neuroinflammatory degeneration. Furthermore, the disintegrin polypeptide obtustatin specifically antagonizes microglial CD49a, suppressing microglial hyperactivation and consequent chronic neuroinflammation, and ultimately ameliorating motor deficits in PD models. Collectively, these findings establish microglial CD49a‐targeted therapy as a novel therapeutic paradigm for PD, positioning obtustatin as a promising clinical candidate with demonstrable translational potential across neuroinflammatory and neurodegenerative disorders.

## Graphical abstract

This study shows that integrin receptor CD49a (*Itga1* gene) is significantly upregulated in hyperactivated microglia and microglia‐specific knockdown of *Itga1* rescues neuroinflammation and neurodegeneration in a chronic Parkinson's disease (PD) model by targeting PGAM5‐mediated mitochondrial dysfunction and NLRP3 activation. Targeted inhibition of CD49a with the disintegrin peptide ameliorates motor deficits, underscoring its therapeutic potential.

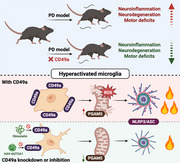

## 1 Introduction

Microglia serve as resident immunomodulatory sentinels of the central nervous system (CNS). Under physiological conditions, they facilitate essential neuroimmune processes, including synaptic pruning, homeostasis maintenance, phagocytic clearance, and intercellular communication [1]. Upon pathological perturbations, microglia undergo dynamic morphological and functional transformations and regulate neuroinflammatory cascades by eliciting heterogeneous molecular responses [2]. Microglia‐mediated neuroinflammation affects neural homeostasis in a dichotomous manner, where timely resolution of acute inflammatory responses is associated with beneficial outcomes, whereas unresolved inflammation propagates neurotoxic cascades [3]. In the initial phase, apoptotic cells, pathological protein accumulation, or endogenous metabolic disturbances trigger microglial activation [4, 5, 6]. Subsequently, activated microglia release anti‐inflammatory mediators, clear pathogens or cellular debris, and restore blood‐brain barrier integrity [7, 8]. Conversely, failed inflammation resolution drives microglial hyperactivation, leading to persistent low‐grade neuroinflammation, ultimately progressing to neurodegeneration, which has been implicated in Parkinson's disease (PD), Alzheimer's disease (AD), Huntington's disease (HD), and amyotrophic lateral sclerosis (ALS) [9, 10, 11, 12]. This pathogenic cascade is mediated by microglia‐derived proteases, cytokines, and oxidative stressors that establish self‐perpetuating neurotoxic feedback loops. Therefore, targeting hyperactivated microglia in the context of chronic neuroinflammation via selective and druggable pathways holds significant promise for addressing current therapeutic challenges in neurodegenerative diseases.

Integrin α1 (CD49a), designated as very late antigen‐1 (VLA‐1), is encoded by the *Itga1* gene. It was initially identified as a marker of terminal T‐cell differentiation, emerging during the late‐stage activation phase following alloantigen or mitogen stimulation [13]. CD49a, a major transmembrane receptor whose primary ligand is collagen IV [14], has been detected in diverse leukocyte populations, including tissue‐resident T cells [15], NK cells [16], and macrophages [17]. Notably, cytokine‐induced CD49a activation triggers outside‐in and inside‐out signaling pathways, mediating cellular adhesion, immune modulation, and inflammatory cascade regulation [18]. CD49a serves as a biomarker for tissue‐resident lymphoid cells, including CD49a^+^ tissue‐resident memory T cells (trmT) [19], and tissue‐resident NK cells (trNK) [20], and critically modulates diverse biological processes, such as antiviral immunity [21, 22], cutaneous immune surveillance [23, 24], tumor growth suppression [25, 26], and joint inflammation [27, 28]. Tissue‐resident macrophages (trM) play pivotal roles in innate immunity while exhibiting significant tissue‐specific heterogeneity. The liver harbors the largest trM population, where CD49a^+^ intrahepatic macrophages demonstrate constitutively elevated baseline production of cytokines, including tumor necrosis factor alpha (TNF‐α), and elevated expression of maturation and activation markers, such as CD86 [17, 29]. In viral myocarditis, signaling protein 7A activated CD49a^+^ cardiac macrophages' production of the proinflammatory cytokines TNF‐α and interleukin‐6 (IL‐6), promoting inflammation and disease progression [30]. The anticancer benefits of chimeric antigen receptor macrophages (CAR‐M) immunotherapy were significantly improved by integrin α1β1‐mediated Fc‐γ receptor I signaling activation, strengthening proinflammatory M1 phenotype‐associated pathways [31]. We hypothesized that CD49a contributes to chronic neuroinflammation in microglia, serving as CNS resident macrophages. However, the dynamics of its expression profile and physiological functions in microglia require further experimental validation.

PD, the second most prevalent neurodegenerative disorder after AD [32], manifests with progressive degeneration of dopaminergic (DA) neurons and pathological accumulation of α‐synuclein‐enriched Lewy bodies within the substantia nigra pars compacta (SNc) [33]. Global epidemiological modeling indicates the PD population will approach 25.2 million by 2050, reflecting a 112% increase from 2021 [34]. Clinically, the prodromal phase of PD presents with non‐motor manifestations, including constipation, depression, rapid eye movement sleep behavior disorder, and mild cognitive impairment [35]. In advanced stages, patients develop cardinal motor symptoms, such as bradykinesia, rigidity, resting tremor, and postural instability [35]. Microglia‐mediated neuroinflammation constitutes a key pathological feature of PD, emerging during the prodromal phase and persisting throughout disease progression until terminal stages [36]. However, it has not been elucidated whether CD49a contributes to PD pathogenesis and progression or represents a viable therapeutic target.

In this study, we aimed to investigate the impact of CD49a in hyperactivated microglia on PD pathogenesis and further elucidate downstream signaling transduction pathways. Utilizing transcriptomic databases, a lipopolysaccharide (LPS) induced PD mouse model, and LPS/interferon‐γ (IFN‐γ) stimulated microglia, we demonstrated that CD49a expression is significantly upregulated in sustained hyperactivated microglia. Knocking CD49a down in a microglia‐specific manner attenuated microglial activation, protected DA neurons from degeneration, and improved motor dysfunction in an 1‐methyl‐4‐phenyl‐1,2,3,6‐tetrahydropyridine (MPTP) induced chronic PD mouse model. Furthermore, transcriptomic profiling of isolated microglia from mouse substantia nigra (SN) coupled with functional validation corroborated these findings and delineated key downstream signaling pathways. Finally, we proposed a peptide‐based therapeutic strategy targeting CD49a, which was designed to accelerate the clinical translation of PD interventions.

## 2 Results

### 2.1 CD49a is Upregulated in Sustained Hyperactivated Microglia

**FIGURE 1:**
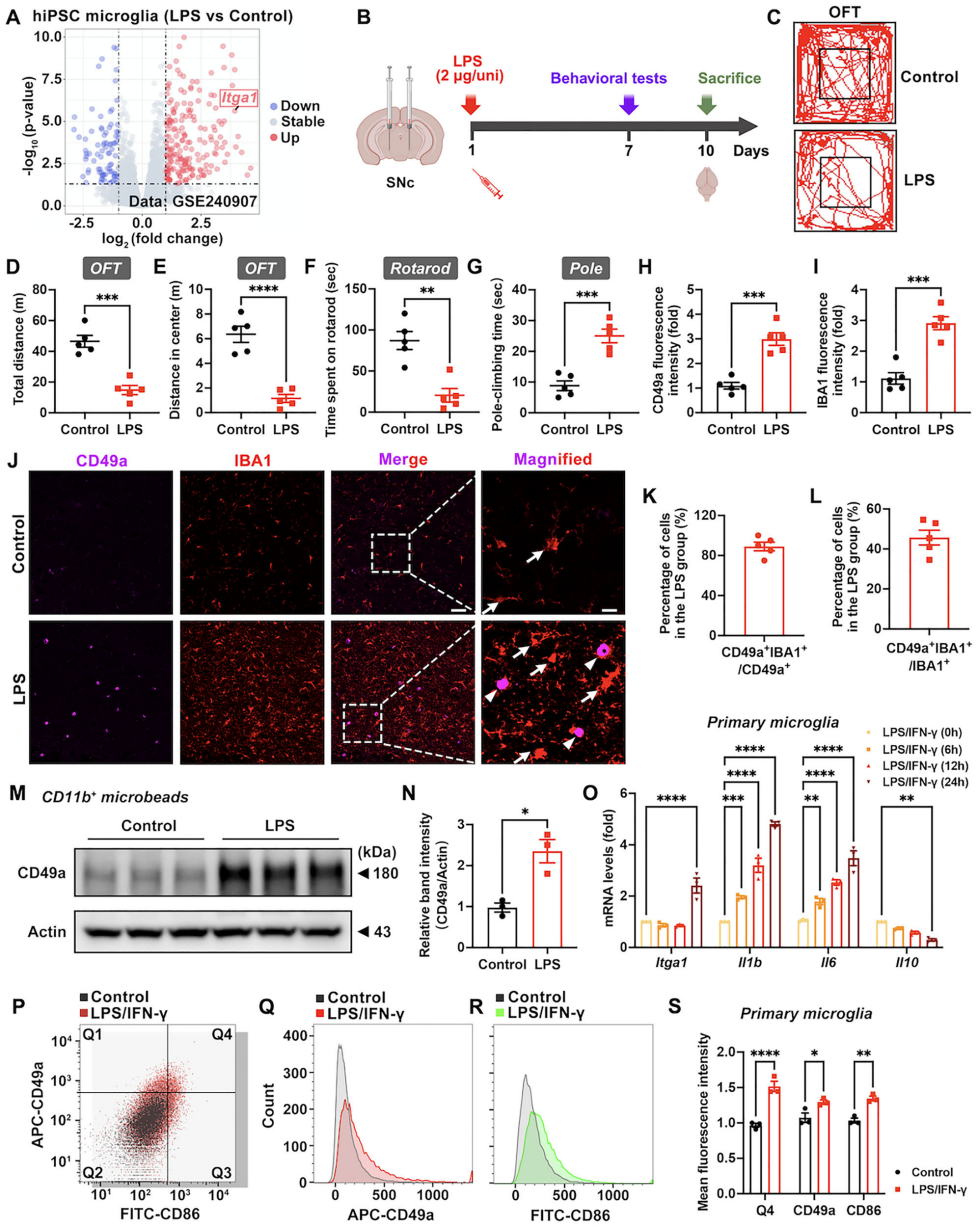
CD49a is upregulated in sustained hyperactivated microglia. (A) Volcano plot illustrating differentially expressed genes (DEGs) comparing lipopolysaccharide (LPS) induced human induced pluripotent stem cell (hiPSC) derived microglia and untreated controls in the GSE240907 dataset. Significantly upregulated (red) and downregulated (blue) genes were defined by thresholds of |log_2_ (fold change)| >1 coupled with Benjamini‐Hochberg adjusted *p*‐value <0.05. *Itga1*'s location has been marked. (B) Schematic diagram of an LPS‐induced PD mouse model, behavioral tests, and brain extraction. (C–E) Travelled trace of mice with quantification of total distance and distance in the center in the open field test (OFT) (*n* = 5). The rectangle in the center represents the central area. (F,G) Analysis of time spent on the rotarod and pole‐climbing time (*n* = 5). (H–J) Immunofluorescence staining with quantification of CD49a and IBA1 expression in the substantia nigra (SN). CD49a, purple fluorescence; IBA1, red fluorescence. White arrows point to IBA1^+^ cells; white arrowheads point to CD49a^+^IBA1^+^ cells. Scale bar: 50 µm for original and 20 µm for magnified images. (K,L) Statistics of the percentage of CD49a^+^IBA1^+^ cells among CD49a^+^ cells and CD49a^+^IBA1^+^ cells among IBA1^+^ cells in the substantia nigra pars compacta (SNc) after an LPS injection (*n* = 5). (M,N) Representative blots with quantification of CD49a protein abundance in microglia (CD11b^+^ microbeads) within the SN (*n* = 3). (O) mRNA transcript abundance of *Itga1*, *Il1b*, *Il6*, and *Il10* in LPS/ interferon‐γ (IFN‐γ) treated primary microglia for distinct durations (0, 6, 12, and 24 h; *n* = 3). The concentrations of LPS and IFN‐γ were 500 ng/mL and 20 ng/mL. (P–S) Representative flow cytometry (FC) images and quantification of CD49a and CD86 protein abundance in primary microglia (*n* = 3). CD49a, APC; CD86, FITC. The Q4 quadrant was defined as the dual‐positive (CD49a^+^ and CD86^+^) cell population. Data are presented as means ± standard error of the mean (SEM) with Student's t‐test (*t*‐test) and two‐way analysis of variance (ANOVA). ^*^ *p* < 0.05, ^**^ *p *< 0.01, ^***^ *p* < 0.001, and ^****^ *p* < 0.0001.

CD49a, a well‐characterized biomarker for tissue‐resident lymphocytes and macrophages in peripheral organs, plays a critical role in inflammation and innate‐ and antigen‐specific immunity [17, 19, 20]. However, its functional implications in microglia, the principal CNS‐resident macrophages, remain elusive. By analyzing The Human Protein Atlas (HPA) (Figure S1A, Supporting Information), we identified higher *Itga1* expression in microglia (60.8 nTPM) than in other major glial cell populations (Figure S1B, Supporting Information). Analysis of differentially expressed genes (DEGs) revealed significant upregulation of *Itag1* in LPS‐induced human induced pluripotent stem cell (hiPSC) derived microglia (Figure 1A), implicating its potential role in hyperactivated microglia and proinflammatory responses. Furthermore, a PD mouse model was constructed by injecting LPS into the SN to examine the contribution of CD49a in neuroinflammation and inflammation‐based motor deficits (Figure 1B). LPS severely impaired the motor performance of mice (Figure 1C–G), damaged DA neurons in the SNc (Figure S1C,D, Supporting Information), and increased the expression of CD49a alongside ionized calcium binding adapter molecule (IBA1, a microglia‐specific marker) in microglia within the SN region (Figure 1H–J,M,N). Further, cell co‐localization analysis indicated that, under LPS stimulation, CD49a was highly expressed primarily in microglia (89.0%), and CD49a was detected in 45.7% of hyperactivated microglia (Figure 1K,L).

Moreover, primary mouse microglia were extracted for in vitro experiments. Different durations (0, 6, 12, and 24 h) of LPS/IFN‐γ treatment affected the mRNA levels of *Itga1* and inflammatory factors (Figure 1O). Among them, LPS/IFN‐γ treatment for 24 h significantly enhanced *Itga1*, *Il1b*, and *Il6* mRNA expression, but reduced *Il10* mRNA expression. Additionally, LPS/IFN‐γ stimulation significantly upregulated both CD49a and CD86 protein expression levels and increased the proportion of CD49a^+^CD86^+^ double‐positive cells (Figure 1P–S). These findings, particularly from the chronic PD mouse model, established CD49a as a defining marker that was distinctly upregulated in hyperactivated microglia.

### 2.2 Microglial Itga1 Knockdown Alleviates Motor Deficits and Pathological Impairments in PD Mice

**FIGURE 2:**
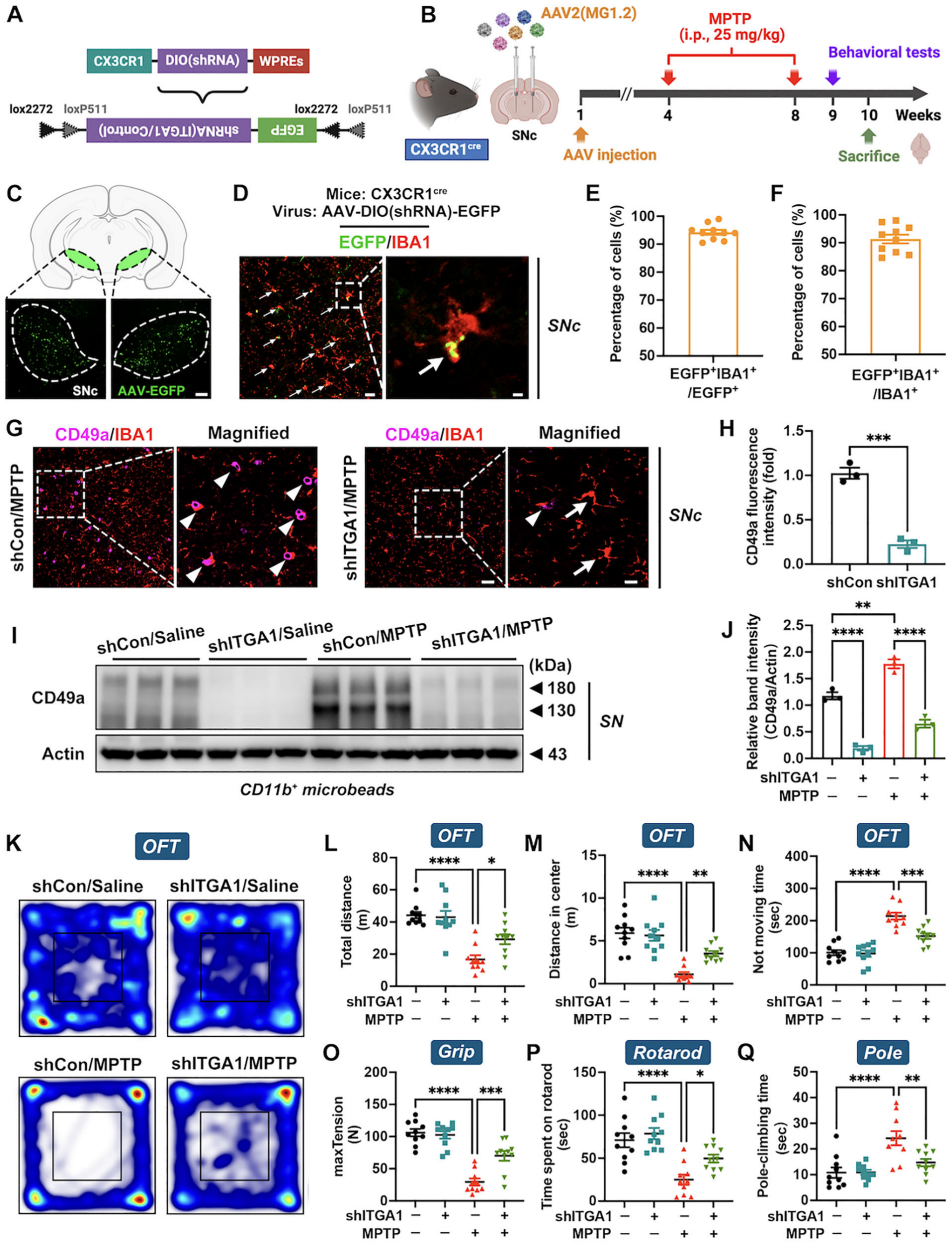
Microglial *Itga1* knockdown alleviates motor deficits in Parkinson's disease (PD) mice. (A) Schematic illustration of the AAV2 (MG1.2) vector construct. The design of the double‐floxed inverse orientation (DIO) cassette is shown in detail. (B) A schematic diagram of an adeno‐associated virus (AAV) injection in CX3CR1^cre^ mice, 1‐methyl‐4‐phenyl‐1,2,3,6‐tetrahydropyridine (MPTP) induced PD model, behavioral tests, and brain extraction. (C) Immunofluorescence staining of AAV‐EGFP in the SNc. AAV‐EGFP, green fluorescence. SNc borders are marked by dashed lines. Scale bar: 100 µm. (D) Co‐localization of EGFP and IBA1 after an AAV microinjection into the SNc. EGFP, green fluorescence; IBA1, red fluorescence. White arrows point to EGFP^+^IBA1^+^ cells. Scale bar: 50 µm for original and 10 µm for magnified images. (E,F) Statistics of the percentage of EGFP^+^IBA1^+^ cells among EGFP^+^ cells and EGFP^+^IBA1^+^ cells among IBA1^+^ cells in the SN after an AAV injection (*n* = 10). (G,H) Representative immunofluorescence staining images with quantification demonstrating *Itga1* knockdown efficiency in the SNc (*n* = 3). CD49a, purple fluorescence; IBA1, red fluorescence. White arrows point to IBA1^+^ cells; white arrowheads point to CD49a^+^IBA1^+^ cells. Scale bar: 50 µm for original and 20 µm for magnified images. (I,J) Representative blots with quantification of CD49a protein abundance in microglia (CD11b^+^ microbeads) within the SN (*n* = 3). (K–N) Travelled trace of mice with quantification of total distance, distance in center, and not moving time in the open field test (OFT) (*n* = 10). (O–Q) Quantification of grip strength test, rotarod test, and pole‐climbing test (*n* = 10). Data are presented as means ± SEM with *t*‐test and one‐way ANOVA. ^*^ *p* < 0.05, ^**^ *p* < 0.01, ^***^ *p* < 0.001, and ^****^ *p* < 0.0001.

To investigate the functional consequences of hyperactivated microglial CD49a in PD pathogenesis, a conditional *Itga1* knockdown mouse, specifically targeting microglia and enabling systematic evaluation of its effects on behavioral phenotypes and neuropathological features, was generated. Specifically, a stereotaxic injection of adeno‐associated virus (AAV) vectors for *Itga1* knockdown (AAV‐shITGA1) or empty vectors (AAV‐shCon) into the SNc of CX3CR1^Cre^ mice was performed [37]. Three weeks post‐injection, an chronic PD model was established by repeated intraperitoneal (i.p.) administration of MPTP for 5 weeks (Figure 2A,B). Cellular co‐localization analysis revealed that AAV‐EGFP primarily transduced microglia (94.2%), with detectable expression in 91.3% of IBA1^+^ cells, indicating high specificity and efficiency in targeting microglia within the SNc (Figure 2C–F). Further, CD49a expression was significantly downregulated in microglia within the SN (Figure 2G–J), confirming successful establishment of the microglia‐specific *Itga1* knockdown model. Subsequently, shITGA1/MPTP group of mice demonstrated marked behavioral improvements relative to the shCon/MPTP group, including increased total distance and central distance in the open field test (OFT), peak tension in the grip strength test, and time spent on the rotarod, as well as reduced not moving time in the OFT and pole‐climbing time (Figure 2K–Q).

**FIGURE 3:**
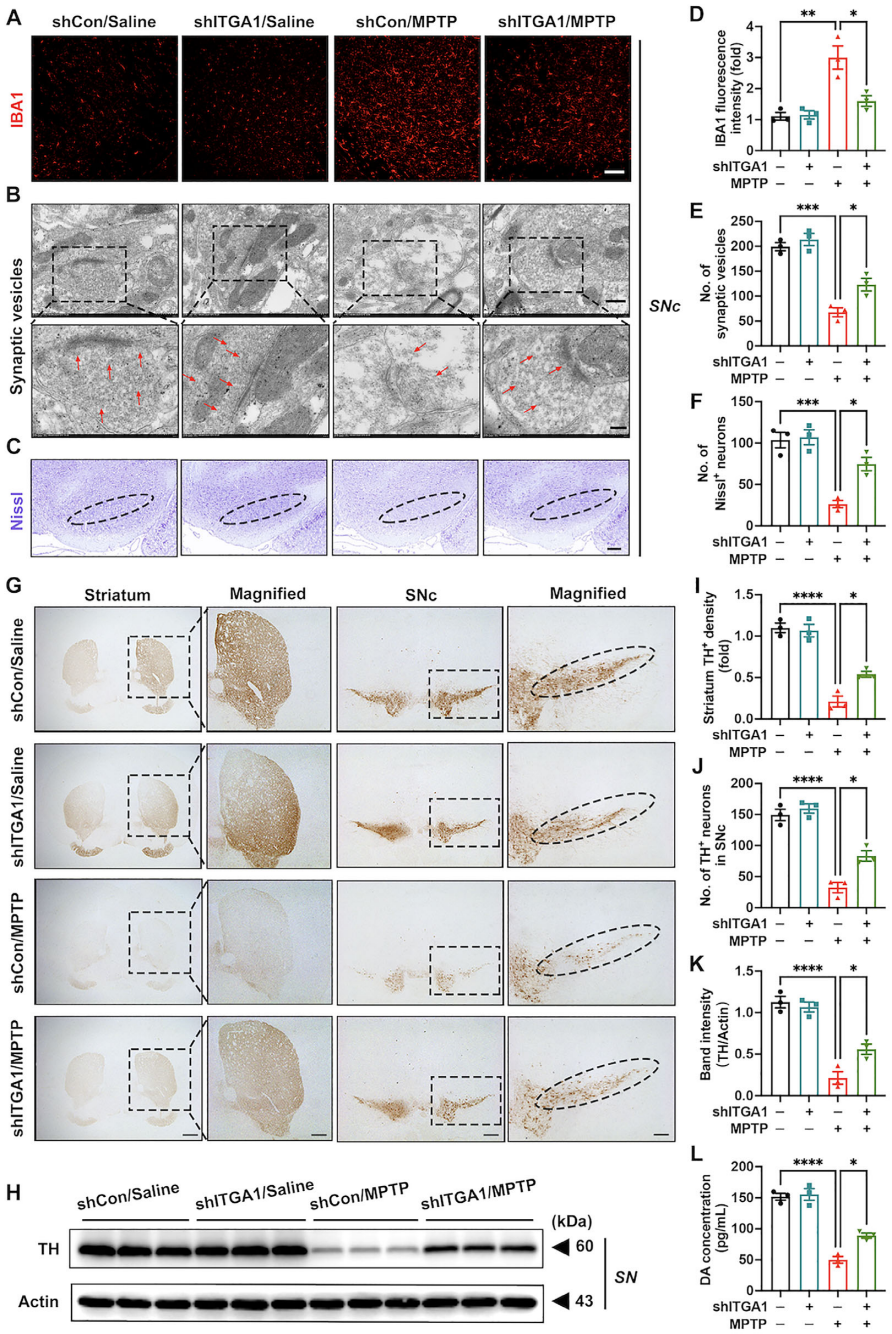
Microglial *Itga1* knockdown alleviates pathological impairments in Parkinson's disease (PD) mice. (A,D) Representative immunofluorescence staining with quantification of IBA1 fluorescence intensity in the SNc (*n* = 3). IBA1, red fluorescence. Scale bar: 100 µm. (B,E) Ultrastructural analysis and quantification of synaptic vesicles in the SNc (*n* = 3). Scale bar: 500 nm for original and 200 nm for magnified images. Red arrows indicate morphological synaptic vesicles. (C,F) Representative Nissl staining images and quantification in the SNc (*n* = 3). Scale bar: 100 µm. SNc borders are marked by ellipses. (G) Representative immunohistochemical staining images in the striatum and SNc. SNc borders are marked by ellipses. Scale bar: 1 mm for original and 200 µm for magnified images in the striatum; 800 µm for original and 100 µm for magnified images in the SNc. (I,J) Quantification of tyrosine hydroxylase (TH)^+^ density in the striatum and TH^+^ neurons in the SNc (*n* = 3). (H,K) Representative blots and quantification of TH expression levels in the SN (*n* = 3). (L) Quantification of dopamine levels in mouse striatal homogenate detected by enzyme‐linked immunosorbent assay (ELISA) (*n* = 3). Data are presented as means ± SEM with *t*‐test and one‐way ANOVA. ^*^ *p* < 0.05, ^**^ *p* < 0.01, ^***^ *p* < 0.001, and ^****^ *p* < 0.0001.

Next, we examined whether microglial *Itga1* knockdown improved key neuropathological features in the PD mouse model. Compared with the shCon/MPTP group, the shITGA1/MPTP group exhibited a significantly reduction in IBA1^+^ fluorescence intensity in the SNc, coupled with an increase in the number of synaptic vesicles and Nissl^+^ cells (Figure 3A–F). Tyrosine hydroxylase (TH) is the rate‐limiting enzyme for dopamine synthesis and is often used to assess the function of DA neurons [38]. Furthermore, TH protein abundance and dopamine content in the striatum as well as number of TH^+^ neurons and TH protein abundance in the SNc were increased (Figure 3G–L). In general, by constructing the microglia‐specific *Itga1* knockdown mouse model, we initially found that genetic inhibition of *Itga1* effectively rescued motor deficits and pathological impairments in PD mice.

### 2.3 Microglial Transcriptome Analysis Reveals Protective Effects of Itga1 Knockdown in a PD Mouse Model

**FIGURE 4:**
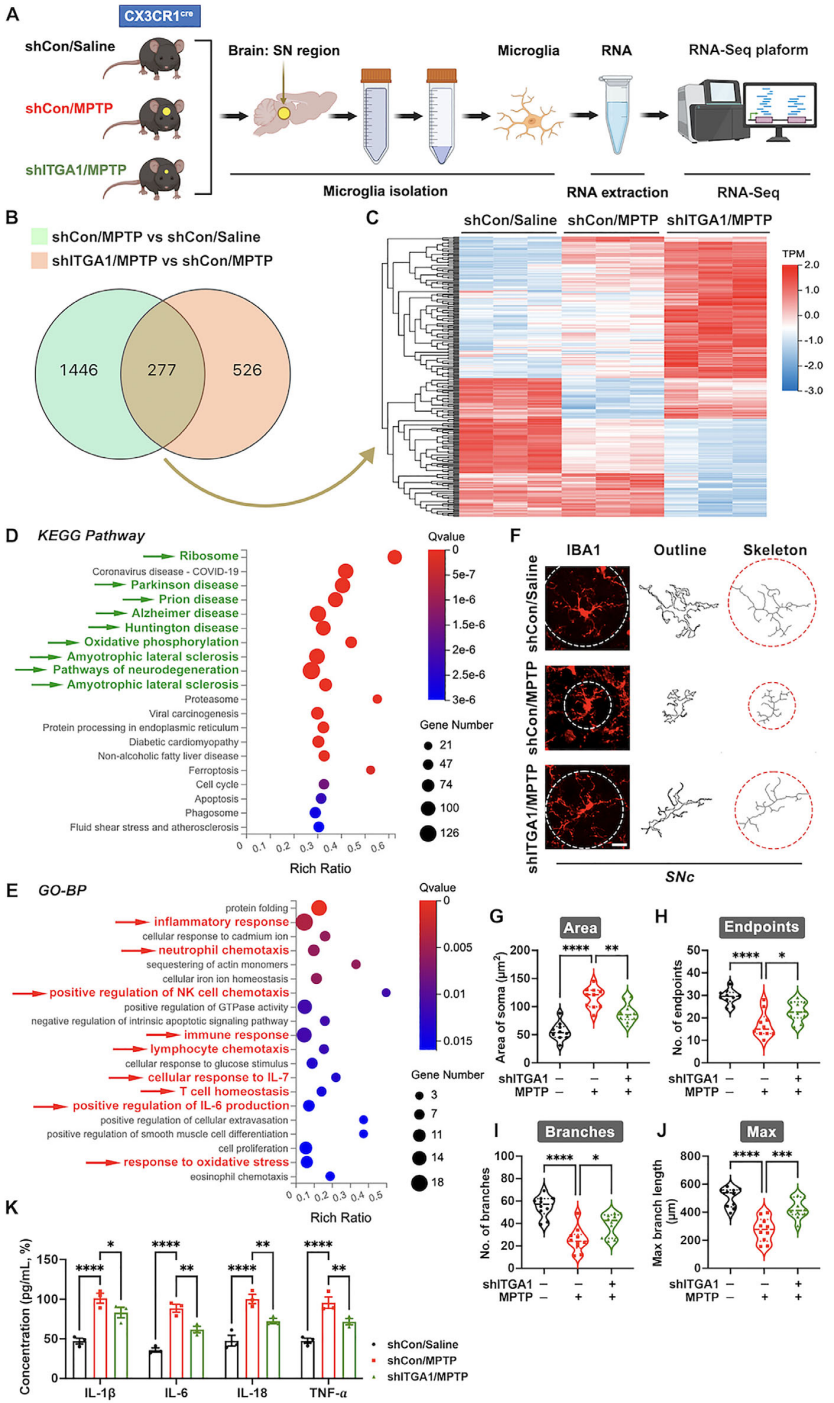
Microglial transcriptome analysis revealed a protective role of *Itga1* knockdown in a Parkinson's disease (PD) mouse model. (A) A schematic diagram of microglial isolation, RNA extraction, RNA‐Seq, and bioinformatic analysis. (B) Venn diagram showing the CDEGs (277) between the “shCon/MPTP vs shCon/Saline” groups (1446) and “shITGA1/MPTP vs shCon/MPTP” groups (526). (C) Hierarchical clustered heatmap of gene expression profiles for the common differentially expressed genes (CDEGs). (D,E) Kyoto Encyclopedia of Genes and Genomes (KEGG) pathway and Gene Ontology Biological Processes (GO‐BP) analysis of the CDEGs. Green arrows point to neurodegenerative KEGG pathways; red arrows point to inflammatory GO‐BP. (F) Representative immunofluorescence images depicting morphological analysis (Outline and Skeleton) of IBA1^+^ microglia in the SNc. Microglia borders are marked by dashed lines. Scale bar: 10 µm. (G–J) Quantification of the soma's area, number of endpoints, number of branches, and maximal branch length in microglia within the SNc (*n* = 10). (K) Quantification of IL‐1β, IL‐6, IL‐18, and TNF‐α concentration levels in mouse striatal homogenate detected by ELISA (*n* = 3). Data are presented as means ± SEM with one‐way and two‐way ANOVA. ^*^ *p* < 0.05, ^**^ *p* < 0.01, ^***^ *p* < 0.001, and ^****^ *p* < 0.0001.

Hyperactivated microglia contribute to PD pathogenesis via multifaceted mechanisms; however, the specific involvement of CD49a in these neuroimmune interactions remains to be elucidated. Therefore, primary microglia were harvested from the SN of shCon/Saline, shCon/MPTP, and shITGA1/MPTP experimental animals, and RNA sequencing (RNA‐Seq) analysis was performed to characterize gene expression and signaling profiles (Figure 4A). Principal component analysis (PCA) demonstrated clustering separation among SN's microglial populations from these three experimental groups (Figure S2A, Supporting Information), indicating that *Itga1* knockdown and MPTP promote significant mRNA profile differences. Volcano plots of DEGs between the “shCon/MPTP versus (vs) shCon/Saline” groups as well as the “shITGA1/MPTP vs shCon/MPTP” groups are shown in Figure S2B,C, Supporting Information. To characterize genes underlying *Itga1* knockdown‐mediated neuroprotection in the PD model, common DEGs (CDEGs) (277) were identified between the “shCon/MPTP vs shCon/Saline” groups (1446) and “shITGA1/MPTP vs shCon/MPTP” groups (526) and are visualized in Figure 4B as a clustered heatmap (Figure 4C). Kyoto Encyclopedia of Genes and Genomes (KEGG) pathway enrichment analysis for CDEGs is shown in Figure 4D. Nine pathways related to neurodegeneration (including PD, AD, HD, and ALS) were ranked among the top enriched pathways. Gene Ontology Biological Processes (GO‐BP) analysis of the CDEGs shown in Figure 4E proved that *Itga1* knockdown can affect signaling pathways related to inflammation (such as inflammatory response, immune response, T‐cell homeostasis, and response to oxidative stress).

Morphological microglial characteristics along with inflammatory mediator levels characterized the degree of neuroinflammation and neurodegeneration [39]. A morphological analysis of microglia in the SNc revealed a decreased area of the soma as well as an increased number of endpoints, number of branches, and maximal branch length in the shITGA1/MPTP group compared to the shCon/MPTP group (Figure 4F–J). Meanwhile, *Itga1* knockdown reduced the striatal concentration of interleukin‐1 beta (IL‐1β), IL‐6, interleukin‐18 (IL‐18), and TNF‐α (Figure 4K). Overall, transcriptome sequencing and experiments have jointly demonstrated that microglial *Itga1* knockdown had a neuroprotective effect in a PD mouse model, mainly manifested in alleviating neuroinflammation.

### 2.4 In Vitro Itga1 Knockdown Attenuates Microglial Hyperactivation

**FIGURE 5:**
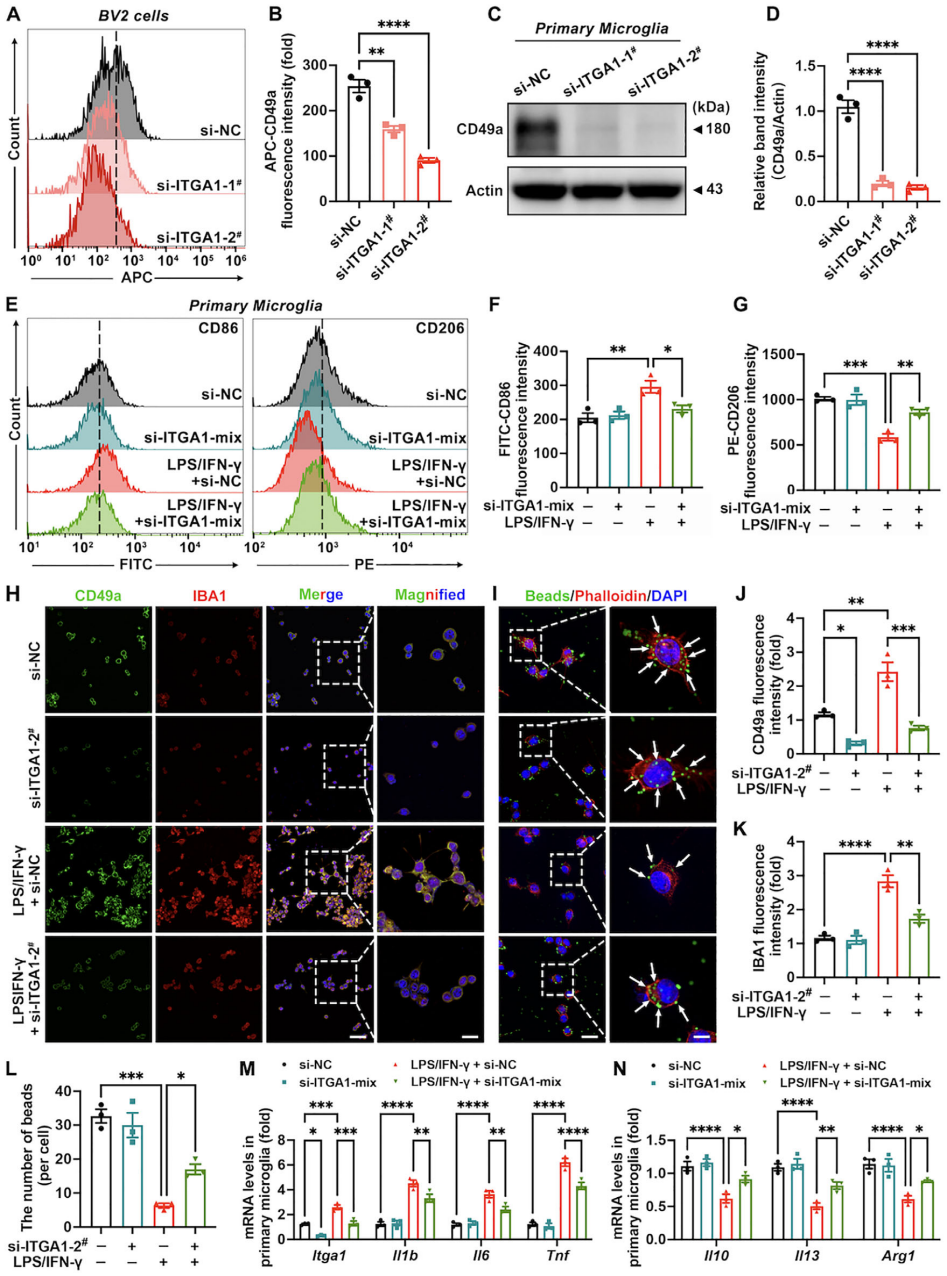
In vitro *Itga1* knockdown attenuates microglial hyperactivation. (A,B) Representative flow cytometry (FC) images with quantitative analysis demonstrating the efficiency of *Itga1* knockdown in BV2 cells using si‐ITGA1‐1^#^ and si‐ITGA1‐2^#^ (*n* = 3). (C,D) Representative blots with quantitative analysis demonstrating the efficiency of *Itga1* knockdown in primary microglia using si‐ITGA1‐1^#^ and si‐ITGA1‐2^#^ (*n* = 3). (E–G) Representative FC images with quantitative analysis of CD86 and CD206 expression in primary microglia (*n* = 3). (H,J,K) Representative immunofluorescence images with quantitative analysis of CD49a and IBA1 expression in BV2 cells (*n* = 3). CD49a, green fluorescence; IBA1, red fluorescence; DAPI, blue fluorescence. Scale bar: 50 µm for original and 20 µm for magnified images. (I,L) Representative immunofluorescence images with quantitative analysis of latex beads' phagocytosis in BV2 cells (*n* = 3). Latex beads, green fluorescence; Phalloidin, red fluorescence; DAPI, blue fluorescence. White arrows point to latex beads phagocytosed by BV2 cells. (M,N) mRNA expression levels of *Itga1*, proinflammatory genes (*Il1b*, *Il6*, and *Tnf*), and anti‐inflammatory genes (*Il10*, *Il13*, and *Arg1*) in primary microglia (*n* = 3). Data are presented as means ± SEM with *t*‐test, one‐way ANOVA, and two‐way ANOVA. ^*^ *p *< 0.05, ^**^ *p* < 0.01, ^***^ *p* < 0.001, and ^****^ *p* < 0.0001.

To further investigate the role of *Itga1* in hyperactivated microglia in vitro, genetic knockdown was performed in both BV2 cells and primary microglia using small interfering RNAs (siRNAs). *Itga1*‐targeting siRNAs (si‐ITGA1‐1^#^ and si‐ITGA1‐2^#^) effectively reduced CD49a protein abundance in BV2 cells and primary microglia (Figure 5A–D). CD86 and CD206 serve as markers indicative of proinflammatory activity and immunoregulatory capacity in microglia, respectively [40]. Compared with the LPS/IFN‐γ + si‐NC group, the si‐ITGA1‐mix (si‐ITGA1‐1^#^ plus 2^#^) downregulated CD86 within primary microglia while upregulating CD206 protein abundance (Figure 5E–G). Similarly, compared with the LPS/IFN‐γ + si‐NC group, si‐ITGA1‐2^#^ reduced the IBA1 expression (Figure 5H,J,K) in BV2 cells and si‐ITGA1‐mix decreased mRNA transcript abundance of *Itga1* and proinflammatory genes (*Il1b*, *Il6*, and *Tnf*) within primary microglia (Figure 5M), indicating inhibition of proinflammatory activity. Cellular phagocytic function is usually characterized by the phagocytic quantity of fluorescent latex beads, which is also a key feature of the tissue‐reparative function of microglia [41]. Our findings demonstrated that si‐ITGA1‐2^#^ significantly increased the number of fluorescent latex beads phagocytosed by BV2 cells (Figure 5I,L) and si‐ITGA1‐mix raised the mRNA levels of anti‐inflammatory genes (*Il10*, *Il13*, and *Arg1*) in primary microglia (Figure 5N). The above results indicated that in vitro knockdown of *Itga1* in microglia inhibited their hyperactivation.

### 2.5 In Vitro Itga1 Knockdown Prevents Hyperactivated Microglia‐mediated Neuronal Death

**FIGURE 6:**
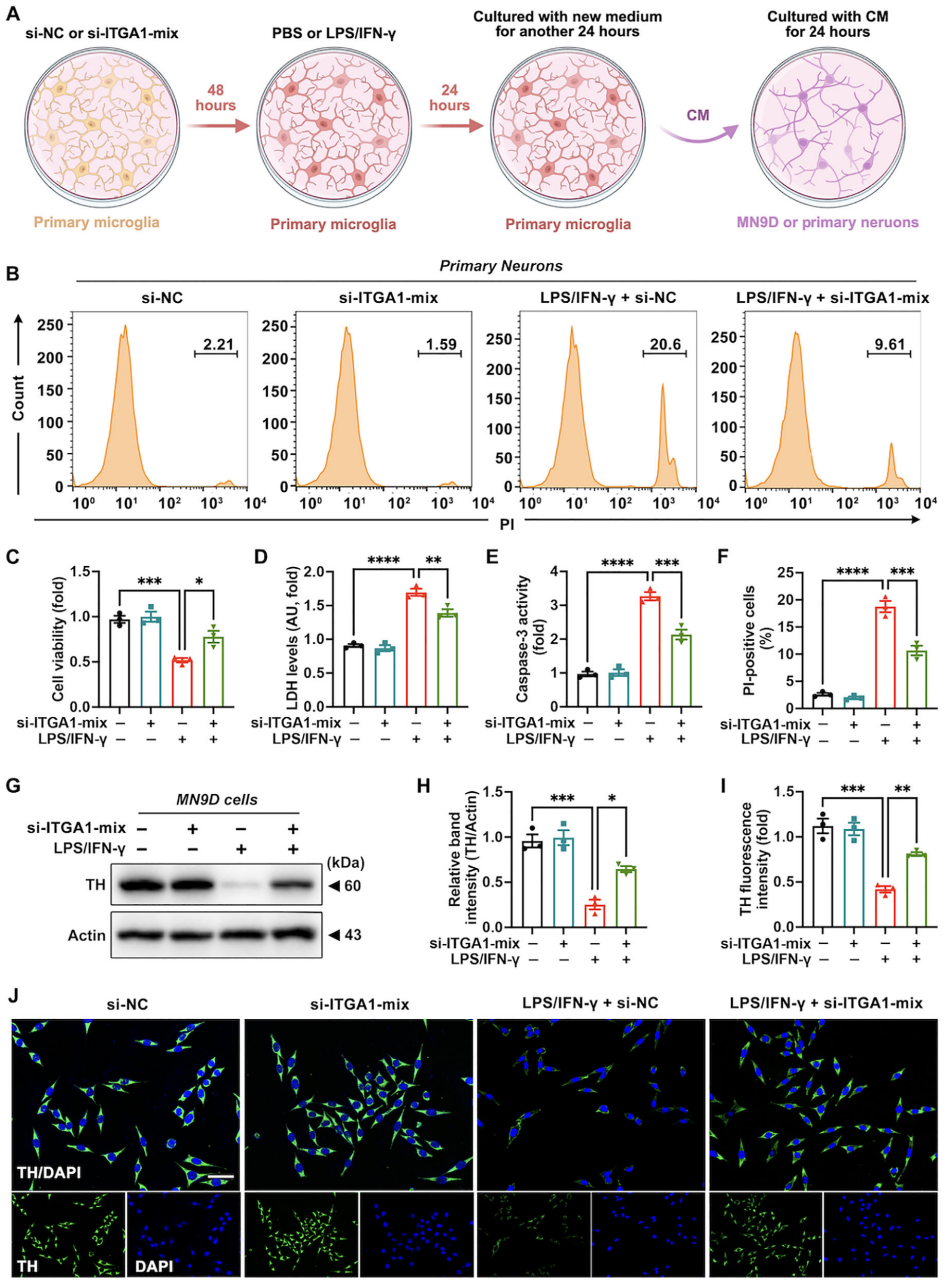
In vitro *Itga1* knockdown prevents hyperactivated microglia‐mediated neuronal death. (A) Experimental workflow depicting conditioned medium (CM) mediated treatment of MN9D cells or primary neurons. Both cell types were incubated with microglial‐derived CM for 24 h. (B,F) Representative flow cytometry (FC) images and quantification of propidium iodide (PI) staining in primary neurons (*n* = 3). (C,D) Quantification of Cell Counting Kit‐8 (CCK8) assay and lactate dehydrogenase (LDH) assay in primary neurons (*n* = 3). (E) Quantification of caspase‐3 activity in primary neurons (*n* = 3). (G,H) Representative blots and quantification of TH expression in MN9D cells (*n* = 3). (I,J) Representative immunofluorescence images and quantification of TH expression in MN9D cells (*n* = 3). TH, green fluorescence; DAPI, blue fluorescence. Scale bar: 50 µm. Data are presented as means ± SEM with one‐way ANOVA. ^*^ *p* < 0.05, ^**^ *p* < 0.01, ^***^ *p* < 0.001, and ^****^ *p* < 0.0001.

Hyperactivated microglia have been shown to secrete diverse inflammatory mediators, which induce neurotoxicity in adjacent neurons [42]. Since our data demonstrated that *Itga1* knockdown suppressed microglial hyperactivation, we hypothesized that this intervention might attenuate microglia‐mediated neurotoxicity. To validate this hypothesis, a mouse DA neuronal cell line (MN9D) and primary neurons were exposed to conditioned medium (CM) obtained from LPS/IFN‐γ‐activated primary microglia following *Itga1* knockdown (Figure 6A). Primary neurons treated with CM from LPS/IFN‐γ‐stimulated primary microglia with *Itga1* knockdown exhibited enhanced cellular survival alongside reduced lactate dehydrogenase (LDH) efflux levels and caspase‐3 activity compared with the model group (Figure 6C–E). The same phenomenon was manifested in primary neurons with propidium iodide (PI) staining. Specifically, primary neurons treated with CM from LPS/IFN‐γ‐stimulated primary microglia with *Itga1* knockdown exhibited fewer PI‐positive cells (Figure 6B,F). Significant rebound of TH protein abundance was detected in MN9D cells treated with CM from LPS/IFN‐γ‐stimulated primary microglia with *Itga1* knockdown compared with the model group (Figure 6G–J). Consequently, microglial *Itga1* knockdown concurrently reduced neuronal death and restored physiological function in DA cell lines.

### 2.6 Microglial Itga1 Knockdown Alleviates Mitochondrial Damage and NLRP3 Inflammasome Activation in a PD Mouse Model via PGAM5

**FIGURE 7:**
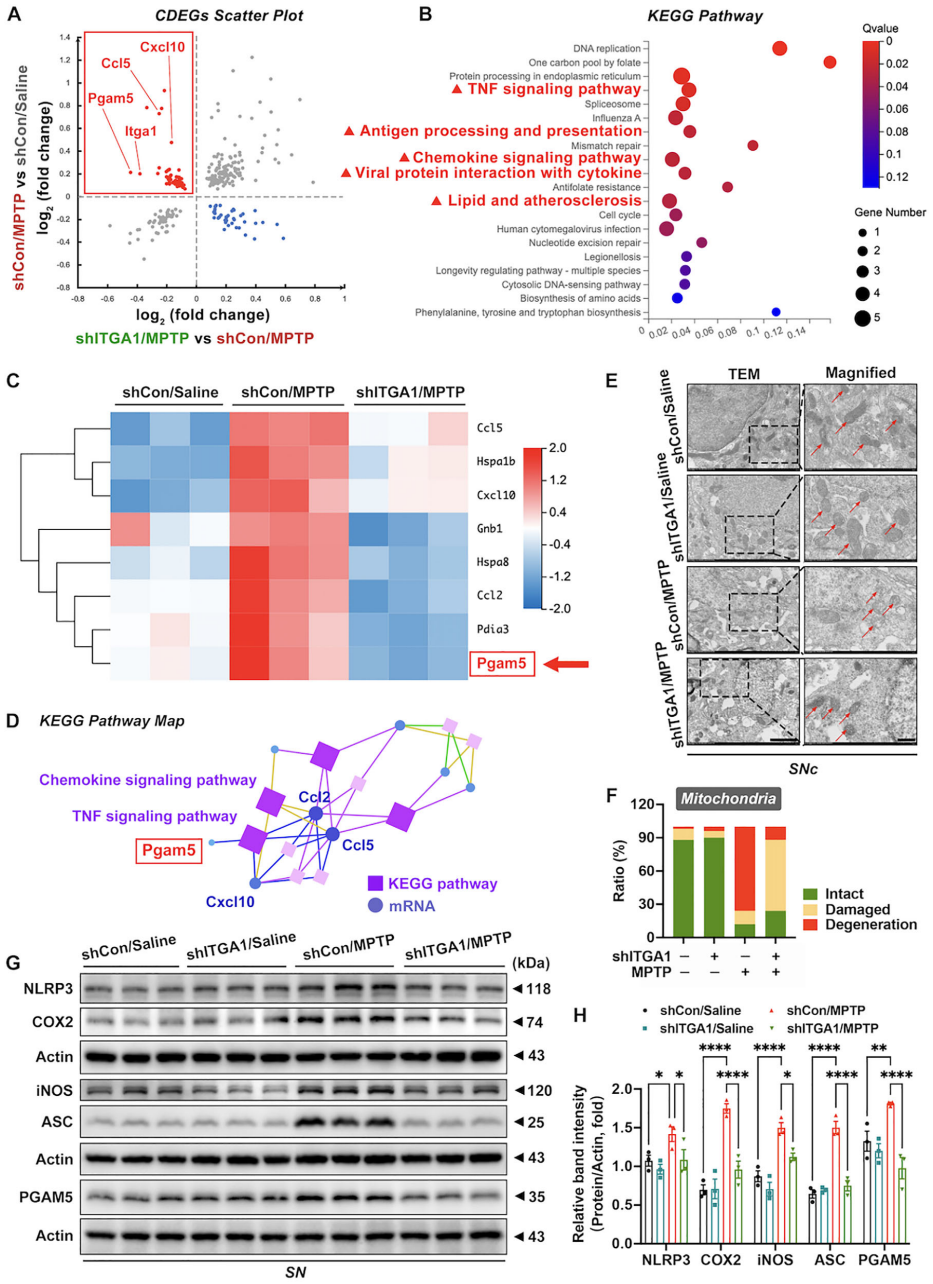
Microglial *Itga1* knockdown alleviates mitochondrial damage and NLRP3 inflammasome activation via PGAM5. (A) Scatter plot highlights common differentially expressed genes (CDEGs) between the “shCon/MPTP vs shCon/Saline” groups and “shITGA1/MPTP vs shCon/MPTP” groups. Red dots indicate the upregulated CDEGs in the first groups (shCon/MPTP vs shCon/Saline) and downregulated CDEGs in the second groups (shITGA1/MPTP vs shCon/MPTP). Blue dots indicate the downregulated CDEGs in the first groups and upregulated CDEGs in the second groups. Key DEGs (*Itga1*, *Pgam5*, *Ccl5*, and *Ccl10*) have been identified and labeled. (B) Kyoto Encyclopedia of Genes and Genomes (KEGG) pathway analysis of CDEGs indicated with red dots from Figure 7A. Red triangles represent inflammatory signaling pathways. (C) Hierarchical clustered heatmap of eight CDEGs in inflammatory signaling pathways. The red arrow points to *Pgam5*. (D) KEGG pathway map of eight CDEGs in inflammatory signaling pathways. Purple rectangles for KEGG pathways; Blue circles for mRNAs. *Pgam5* is identified in red. (E,F) Ultrastructural images and quantification of mitochondria in the SNc (*n* = 5). Scale bar: 500 nm for original and 200 nm for magnified images. Red arrows represent mitochondria. The green, yellow, and red in the statistical chart represent normal, damaged, and degenerated mitochondria, respectively. (G,H) Representative blots and quantification of NLRP3, COX2, iNOS, ASC, and PGAM5 protein expression levels in the SN (*n* = 3). Data are presented as means ± SEM with one‐way and two‐way ANOVA. ^*^ *p* < 0.05, ^**^ *p* < 0.01, ^***^ *p* < 0.001, and ^****^ *p* < 0.0001.

Next, the molecular signaling pathways via which CD49a mediates microglial hyperactivation were investigated. Given the established upregulation of neuroinflammatory genes in PD pathogenesis, we specifically analyzed CDEGs that were significantly elevated in “shCon/MPTP vs shCon/Saline” comparisons and concurrently attenuated in “shITGA1/MPTP vs shCon/MPTP” comparisons (red scatter dots in Figure 7A). A KEGG pathway enrichment analysis of the above‐mentioned CDEGs identified five significantly enriched inflammatory pathways for subsequent analysis, as shown in the red triangle (Figure 7B). Our analysis focused on CDEGs associated with the identified inflammatory pathways and involved comprehensive KEGG pathway mapping and a systematic literature review to elucidate their functional significance (Figure 7C,D). Ultimately, we focused on phosphoglycerate mutase family 5 (PGAM5), a mitochondrial phosphatase, as a key regulator of multiple pathological processes in PD, including mitochondrial homeostasis, programmed cell death pathways, inflammation, and immune regulation [43, 44, 45]. An ultrastructural transmission electron microscopy (TEM) analysis demonstrated that microglial *Itga1* knockdown significantly reduced the prevalence of morphologically damaged and degenerated mitochondria in the SNc (Figure 7E,F). Subsequent experimental validation revealed that MPTP administration significantly upregulated PGAM5 expression and proinflammatory markers (iNOS and COX2) (shCon/MPTP vs shCon/Saline), while microglial *Itga1* knockdown effectively attenuated these changes (shITGA1/MPTP vs shCon/MPTP) (Figure 7G,H). Previous investigations have demonstrated that PGAM5 facilitates NLR family pyrin domain containing 3 (NLRP3) inflammasome activation, consequently amplifying the secretion of proinflammatory cytokines, including IL‐1β and IL‐18 [46, 47]. Our investigation specifically focused on NLRP3 and apoptosis‐associated speck‐like protein containing a caspase recruitment domain (ASC), essential structural components of the NLRP3 inflammasome complex that co‐assemble during inflammasome activation. Notably, microglial *Itga1* knockdown significantly attenuated the MPTP‐induced upregulation of both NLRP3 and ASC proteins in PD mice (Figure 7G,H). Collectively, our data established that microglial *Itga1* knockdown attenuated mitochondrial pathology and suppressed NLRP3‐ASC inflammasome activation in the SN of a PD mouse model. PGAM5 emerged as a plausible mechanistic intermediary, which should be further investigated in in vitro experiments.

### 2.7 In Vitro Microglial Itga1 Knockdown Attenuates Mitochondrial Damage and Inflammasome Activation via PGAM5

**FIGURE 8:**
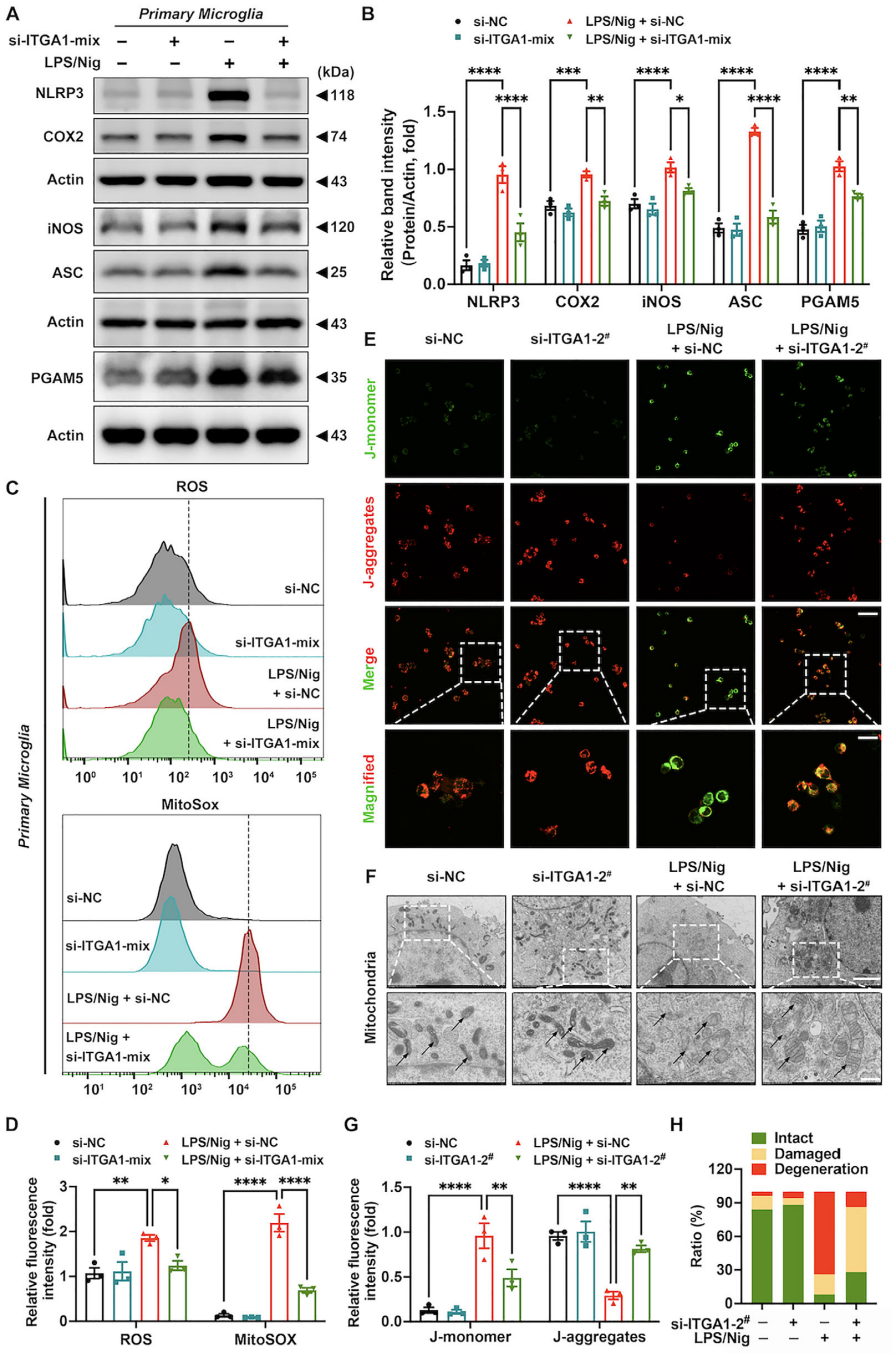
In vitro microglial *Itga1* knockdown alleviates mitochondrial damage and NLRP3 inflammasome activation via PGAM5. (A,B) Representative blots and quantification of NLRP3, COX2, iNOS, ASC, and PGAM5 protein expression levels in primary microglia (*n* = 3). (C,D) Representative flow cytometry (FC) images and quantification of intracellular and mitochondrial reactive oxygen species (ROS) generation in primary microglia (*n* = 3). (E,G) Representative fluorescence images and quantification of mitochondrial Δψm in BV2 cells (*n* = 3). J‐monomers, green fluorescence; J‐aggregates, red fluorescence. Scale bar: 50 µm for original and 20 µm for magnified images. (F,H) Ultrastructural images and quantification of mitochondria in BV2 cells (*n* = 5). Scale bar: 500 nm for original and 200 nm for magnified images. Black arrows represent mitochondria in BV2 cells. The green, yellow, and red in the statistical chart represent normal, damaged, and degenerated mitochondria, respectively. Data are presented as means ± SEM with one‐way and two‐way ANOVA. ^*^ *p* < 0.05, ^**^ *p* < 0.01, ^***^ *p* < 0.001, and ^****^ *p* < 0.0001.

An LPS‐primed, nigericin‐stimulated microglial activation model was employed to investigate the effect of *Itga1* knockdown on PGAM5‐mediated mitochondrial damage and inflammasome activation in vitro. Nigericin, a potent NLRP3 inflammasome activator, was used to induce robust inflammasome assembly following LPS priming [48]. Consistent with our in vivo findings, *Itga1* knockdown in primary microglia significantly attenuated the upregulation of iNOS, COX2, PGAM5, NLRP3, and ASC in the LPS/nigericin (LPS/Nig) model group (Figure 8A,B). Given the role of PGAM5 as a mitochondrial inner membrane protein and phosphatase critically involved in mitochondrial homeostasis [49], key functional parameters of microglial mitochondria were assessed systematically. *Itga1* knockdown significantly reduced cellular and mitochondrial reactive oxygen species (ROS) in primary microglia (Figure 8C,D) and increased mitochondrial membrane potential (Δψm) in BV2 cells (Figure 8E,G). Ultrastructural TEM analysis revealed that *Itga1* knockdown significantly reduced the prevalence of morphologically damaged and degenerated mitochondria in BV2 cells (Figure 8F,H). Consistent with these findings, Hoechst staining demonstrated a marked attenuation of mitochondrial damage‐associated cell death (Figure S3, Supporting Information), cumulatively suggesting that CD49a derived microglial hyperreactivity by inducing mitochondrial dysfunction and inflammatory cell death, primarily via regulation of the PGAM5‐NLRP3 inflammasome axis.

### 2.8 Microglial PGAM5 Overexpression Reverses Anti‐inflammatory Effects and Mitochondrial Homeostasis Mediated by Itga1 Knockdown

**FIGURE 9:**
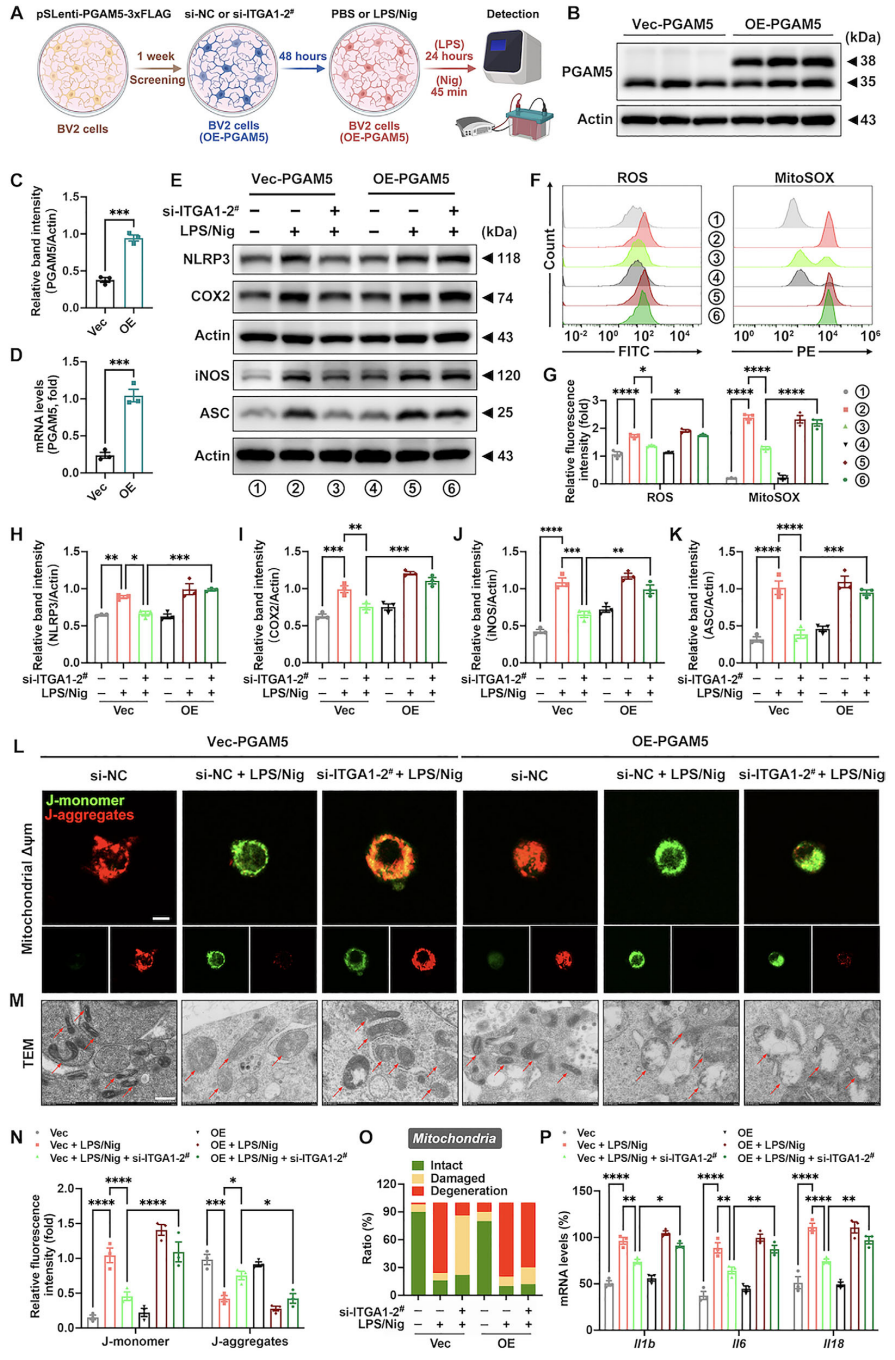
Microglial PGAM5 overexpression reverses anti‐inflammatory effects and mitochondrial homeostasis mediated by *Itga1* knockdown. (A) Experimental workflow depicting the experimental procedure involving the establishment of stable OE‐PGAM5 BV2 cells, transfection with si‐ITGA1‐2^#^, LPS/Nig stimulation protocol, and detection. (B,C) Representative blots and quantification of PGAM5 protein expression levels in OE‐PGAM5 or Vec‐PGAM5 BV2 cells (*n* = 3). (D) The mRNA expression levels of PGAM5 in OE‐PGAM5 or Vec‐PGAM5 BV2 cells (*n* = 3). (E,H–K) Representative blots and quantification of NLRP3, COX2, iNOS, ASC, and PGAM5 protein expression levels in OE‐PGAM5 or Vec‐PGAM5 BV2 cells (*n* = 3). (F,G) Representative FC images and quantification of both cellular and mitochondrial ROS levels in OE‐PGAM5 or Vec‐PGAM5 BV2 cells (*n* = 3). (L,N) Representative fluorescence images and quantification of mitochondrial Δψm in OE‐PGAM5 or Vec‐PGAM5 BV2 cells (*n* = 3). J‐monomers, green fluorescence; J‐aggregates, red fluorescence. Scale bar: 10 µm. (M,O) Ultrastructural images and quantification of mitochondria in OE‐PGAM5 or Vec‐PGAM5 BV2 cells (*n* = 5). Red arrows represent mitochondria. Scale bar: 200 nm. The green, yellow, and red in the statistical chart represent respectively normal, damaged, and degenerated mitochondria. (P) The mRNA transcript abundance of proinflammatory genes (*Il1b*, *Il6*, and *Il18*) in OE‐PGAM5 or Vec‐PGAM5 BV2 cells (*n* = 3). Data are represented as means ± SEM with *t*‐test, one‐way ANOVA, and two‐way ANOVA. ^*^ *p* < 0.05, ^**^ *p* < 0.01, ^***^ *p* < 0.001, and ^****^ *p* < 0.0001.

To determine whether PGAM5 serves as a critical downstream effector of CD49a in neuroinflammatory regulation and mitochondrial homeostasis, we constructed stable BV2 cell lines with PGAM5 overexpression (OE‐PGAM5) or empty vector control (Vec‐PGAM5) through lentiviral transduction followed by puromycin selection (Figure 9A). The successful generation was validated at both the transcriptional and translational levels (Figure 9B–D). Notably, while *Itga1* knockdown suppressed the expression of inflammatory mediators (iNOS and COX2) and inflammasome components (NLRP3 and ASC) in LPS/Nig‐stimulated microglia, OE‐PGAM5 effectively abrogated this protective effect (Figure 9E,H–K). Furthermore, OE‐PGAM5 increased cellular and mitochondrial ROS levels (Figure 9F,G) and decreased mitochondrial Δψm in BV2 cells (Figure 9L,N) compared with *Itga1* knockdown. OE‐PGAM5 reversed the mitochondrial ultrastructural changes induced by *Itga1* knockdown, primarily evidenced by a significant increase in the prevalence of morphologically damaged and degenerated mitochondria in BV2 cells (Figure 9M,O). Meanwhile, OE‐PGAM5 increased the mRNA transcript abundance of proinflammatory genes (*Il1b*, *Il6*, and *Il18*) in BV2 cells compared to *Itga1* knockdown (Figure 9P). Utilizing an OE‐PGAM5 microglial model to reconstitute downstream signaling, PGAM5's hierarchical core function in CD49a‐mediated neuroinflammatory cascades was confirmed experimentally.

### 2.9 Microglial PGAM5 Overexpression Aggravates Neuronal Death

To further elucidate the neurotoxic effects of microglial OE‐PGAM5 on DA neurons and delineate its critical role in CD49a‐mediated pathways, MN9D cells or primary neurons were treated with BV2 cell‐derived CM that had undergone the experimental procedures outlined in Figure S4A, Supporting Information. CM from OE‐PGAM5 BV2 cells significantly abrogated the neuroprotective effects mediated by microglial *Itga1* knockdown, as evidenced by reversal of enhanced neuronal viability (Figure S4C, Supporting Information) and restoration of LDH release (Figure S4D, Supporting Information) and caspase‐3 activity (Figure S4E, Supporting Information). Consistent with these findings, CM from OE‐PGAM5 BV2 cells markedly increased the number of PI‐positive primary neurons (Figure S4B,F, Supporting Information), further confirming its detrimental impact on neuronal survival. CM from OE‐PGAM5 BV2 cells significantly reduced the increased TH expression in MN9D cells observed with microglial *Itga1* knockdown (Figure S4G–J, Supporting Information). Collectively, these data implicated PGAM5 as a master regulator of CD49a‐driven microglial hyperactivation, culminating in neuronal death and DA dysfunction, establishing its role as a critical nexus between neuroinflammation and neuronal homeostasis.

### 2.10 Obtustatin Specifically Binds to CD49a

**FIGURE 10:**
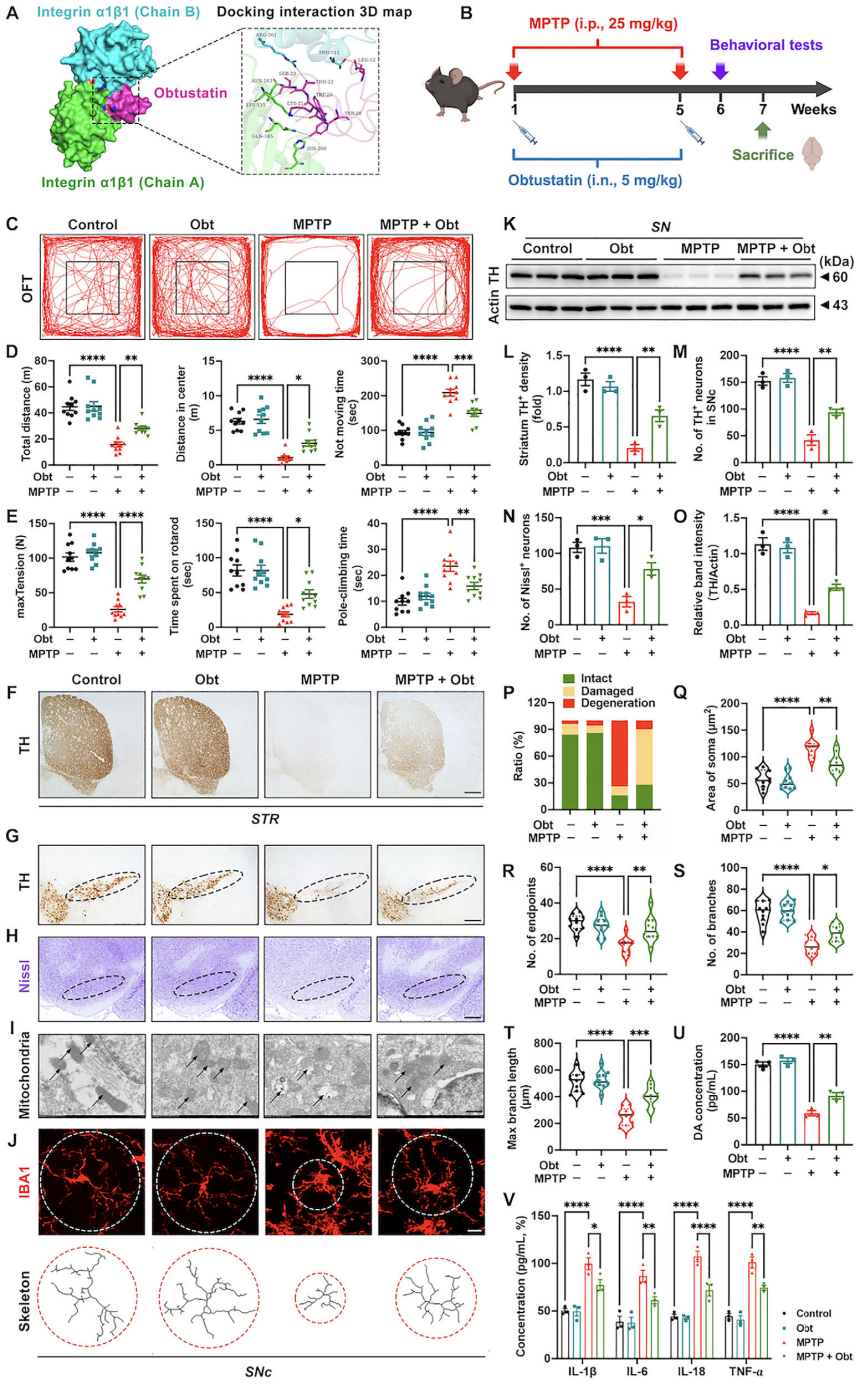
Pharmacological inhibition of CD49a relieves neuroinflammation and improves motor impairments in a Parkinson's disease (PD) mouse model. (A) Molecular docking analysis of obtustatin (PDB: 1MPZ) with integrin α1β1 (PDB: 1QC5) (left) and the potential interaction binding site (right). Obt, obtustatin; Green part, integrin α1β1 (Chain A); blue part, integrin α1β1 (Chain B); magenta part, obtustatin. (B) Experimental design involving obtustatin treatment (i.n.; 5 mg/kg) in an 1‐methyl‐4‐phenyl‐1,2,3,6‐tetrahydropyridine (MPTP) induced chronic PD mouse model (i.p.; 25 mg/kg). Obtustatin and MPTP were administered twice weekly for 5 weeks. Obtustatin was administered in the morning and MPTP in the afternoon, with an interval of 6 h. Behavioral tests were performed in Week 6. The experimental animals were sacrificed in Week 7. (C) Travelled trace of mice in the open field test (OFT). (D,E) Quantification of the total distance, distance in center, and not moving time in the OFT as well as quantification of the grip strength test, rotarod test, and pole‐climbing test (*n* = 10). (F,L) Representative immunohistochemical staining images and quantification of TH^+^ density in the striatum. Scale bar: 200 µm. (G,M) Representative immunohistochemical staining images and quantification of TH^+^ neurons in the SNc (*n* = 3). Borders of the SNc are shown by ellipses. Scale bar: 100 µm. (H,N) Representative Nissl staining images and quantification in the SNc (*n* = 3). Scale bar: 100 µm. Borders of the SNc are shown by ellipses. (I,P) Ultrastructural images and quantification of mitochondria in the SNc (*n* = 5). Scale bar: 200 nm. Black arrows represent mitochondria. The green, yellow, and red in the statistical chart represent normal, damaged, and degenerated mitochondria, respectively. (J) Representative immunofluorescence images depicting morphological analysis (Skeleton) of IBA1^+^ microglia in the SNc of mice. Microglia borders are marked by dashed lines. Scale bar: 10 µm. (K,O) Representative blots and quantification of TH expression levels in the SN (*n* = 3). (Q–T) Quantification of soma's area, number of endpoints, number of branches, and maximal branch length in SNc microglia. (*n* = 10). (U) Quantification of dopamine concentrations in striatal tissue homogenates measured using ELISA (*n* = 3). (V) Quantification of IL‐1β, IL‐6, IL‐18, and TNF‐α concentration levels in mouse striatum homogenate detected by ELISA (*n* = 3). Data are presented as means ± SEM with *t*‐test and one‐way ANOVA. ^*^ *p* < 0.05, ^**^ *p* < 0.01, ^***^ *p* < 0.001, and ^****^ *p* < 0.0001.

Given the established proinflammatory role of CD49a in PD, its targeted pharmacological inhibition represents a promising therapeutic strategy to mitigate neuroinflammation. Obtustatin, a KTS (Lys‐Thr‐Ser) disintegrin inhibitor, is the most potent and selective currently available polypeptide antagonist of integrin α1β1, with established industrial‐scale production protocols and proven in vivo therapeutic efficacy [50, 51]. To validate the specific binding between obtustatin and CD49a, cell adhesion assays and antibody competition experiments were performed. At concentrations of 1, 2.5, and 5 µm, obtustatin not only inhibited microglial adhesion to collagen IV (a known CD49a ligand) but also competitively displaced anti‐CD49a antibody binding, demonstrating a dose‐dependent efficacy in both functional and binding assays (Figure S5A–D, Supporting Information). Molecular docking analysis revealed that obtustatin adopted a bent, flexible conformation that inserted into the interface between two chains of integrin α1β1 (Figure 10A (left); Figure S5E, Supporting Information). Critical binding interactions were identified between obtustatin residues (Trp‐20, Tyr‐28, Thr‐22, Leu‐12, Ser‐23, and Lys‐21) and integrin α1β1 (Chain A) residues (Gln‐165, Lys‐135, Asn‐163, and His‐200) as well as integrin α1β1 (Chain B) residues (Arg‐361 and Thr‐512), forming distinct interaction hot spots (Figure 10A (right)). These findings indicated that obtustatin's engagement with CD49a primarily spanned both subunits, potentially enhancing dimer stabilization. The binding mode featured non‐covalent interactions (hydrogen bonding), indicating reversible binding that was physiologically suitable for modulating biological activity.

Building upon the molecular docking results, 100‐ns molecular dynamics simulations (MD) were conducted to comprehensively evaluate the binding stability and interaction dynamics of the protein complex. Systematic analysis of key parameters, including root‐mean‐square deviation (RMSD), root‐mean‐square fluctuation (RMSF), radius of gyration (Rg), solvent‐accessible surface area (SASA), and hydrogen bonding patterns, demonstrated that the complex maintained stable and compact conformational states throughout the simulation, with a well‐preserved hydrogen bond network (Figure S5F–K, Supporting Information). Based on a Gibbs energy landscape that revealed a thermodynamically stable lowest‐energy conformation of the protein complex (as evidenced by RMSD and Rg values; Figure S5L,M, Supporting Information), we performed a per‐residue energy decomposition diagram to quantify the contribution of hot residues (Figure S5N, Supporting Information). Furthermore, MM/PBSA calculations were performed on the 20‐ns equilibrated trajectory and demonstrated favorable binding thermodynamics (Δ*G*_total_ = −62.07 kcal/mol), indicating spontaneous and stable complex formation (Table S2, Supporting Information). These computational results collectively validated obtustatin's high‐affinity binding potential to integrin α1β1, supporting its development as either a targeted therapeutic or functional peptide lead.

### 2.11 Pharmacological Inhibition of CD49a Relieves Neuroinflammation and Improves Motor Deficits in a PD Mouse Model

Dose‐optimization studies (1, 2.5, and 5 mg/kg) identified 5 mg/kg intranasal (i.n.) administration of obtustatin as the optimal therapeutic dose, demonstrating maximal efficacy in both ameliorating motor deficits in a PD mouse model and suppressing microglial activation (Figure S6, Supporting Information) without significant hepatorenal and immunological toxicity (Figure S7, Supporting Information). Therefore, 5 mg/kg obtustatin was selected as the optimal dose for subsequent experimental validation (Figure 10B). Obtustatin significantly attenuated MPTP‐induced motor deficits as demonstrated by improved performance in the OFT, grip strength, pole‐climbing, and rotarod tests (Figure 10C–E). Specifically, 5 mg/kg obtustatin improved motor function of PD mice by increasing the total distance and central distance in the OFT, peak tension in the grip strength test, and time spent on the rotarod, as well as reducing not moving time in the OFT and pole‐climbing time. These findings demonstrated the potential benefits of obtustatin for treating motor dysfunction.

Next, we evaluated the core pathological manifestations in the PD mouse model. Obtustatin significantly increased TH expression (Figure 10F,L) and dopamine content (Figure 10U) in the striatum as well as the number of TH^+^ (Figure 10G,M) and Nissl^+^ (Figure 10H,N) neurons and TH expression (Figure 10K,O) in the SN compared with the model group. Mitochondrial dysfunction is a well‐documented pathological hallmark of PD and is consistently observed in both preclinical models and patient‐derived samples [52, 53]. Obtustatin reversed MPTP‐induced mitochondrial ultrastructural changes, primarily evidenced by a significant decrease in the prevalence of morphologically damaged and degenerated mitochondria in the SNc (Figure 10I,P). For the morphological analysis of SNc microglia, obtustatin reduced the soma's area but increased the number of endpoints, number of branches, and maximal branch length (Figure 10J,Q–T). Meanwhile, obtustatin reduced IL‐1β, IL‐6, IL‐18, and TNF‐α concentrations in the striatum (Figure 10V). Collectively, these findings demonstrated that obtustatin significantly attenuated neuroinflammation and protected DA neurons in a PD mouse model.

## 3 Discussion

Microglia, which are the principal CNS resident immune cells, play a pivotal role in the pathophysiology of PD via secretion of proinflammatory cytokines [42]. Preventing chronic microglial hyperactivation can effectively treat PD, but better therapeutic targets need to be explored. CD49a, an α‐integrin subunit abundantly expressed on the cell membrane of diverse tissue‐resident immune cells, is a well‐documented mediator of persistent inflammatory cascades across multiple organ systems [17, 19, 20]. We demonstrated the dynamic expression and function of CD49a in microglia. LPS upregulated CD49a microglial expression, while conditional microglial *Itga1* knockdown in the SNc suppressed persistent microglial hyperactivation, attenuated hyperactivated microglia‐mediated DA death, and markedly improved motor deficits in a PD mouse model. Collectively, our data established CD49a‐mediated microglial hyperactivation as a sustained neuroinflammatory driver of PD pathogenesis, identifying it as a tractable pharmacological target for disease modification.

Since microglial hyperactivation is a hallmark of chronic neuroinflammation [3], we employed complementary approaches to dissect the indispensable role of CD49a in sustaining pathogenic microglial states and driving PD progression. We found that, even after an MPTP injection, the number of IBA1^+^ cells in the SN of mice with microglial *Itga1* knockdown was significantly reduced. Microglial morphology is characterized by dynamic structural reorganization, shifting from a highly ramified surveillance phenotype under homeostasis to an amoeboid morphology upon activation, reflecting functional state transitions [54, 55]. Our research indicated that *Itga1* knockdown reduced more amoebic microglia, with smaller cell bodies and longer branches. Upon pathological hyperactivation, proinflammatory activity and immunoregulatory capacity in microglia profoundly reshape the neural microenvironment via dysregulated cytokine secretion, critically influencing PD progression [56]. Our findings demonstrated that *Itga1* knockdown significantly attenuated microglial proinflammatory responses, evidenced by downregulated CD86 expression and reduced secretion of inflammatory mediators, and concurrently enhanced tissue‐reparative function via increased CD206 expression and elevated anti‐inflammatory factor secretion, ultimately enhancing microglial phagocytic capacity under LPS/IFN‐γ co‐stimulation. We further stimulated primary microglia with IL‐4 and assessed *Itga1* transcriptional dynamics. Numerous studies have demonstrated that IL‐4 robustly polarizes microglia towards an anti‐inflammatory phenotype with enhanced neurotrophic functions [57, 58]. We substantiated these findings and further discovered that chronic IL‐4 exposure significantly downregulated *Itga1* expression, whereas acute stimulation produced negligible effects (Figure S1E, Supporting Information). LPS/IFN‐γ or IL‐4 stimulation temporally regulated CD49a expression, demonstrating its correlation with microglial phenotypic switching via delayed‐response mechanisms and warranting rigorous experimental validation.

We also validated the essential role of the neural inflammatory microenvironment, which was facilitated by aberrantly activated microglia, in exacerbating DA neurons degeneration and movement impairment. Hyperresponsive microglia have been shown to disrupt neural‐immune homeostasis, thereby directly impairing neuronal viability via dysregulated neuroinflammatory cascades [42]. Using RNA‐Seq and subsequent bioinformatics analysis, our study demonstrated that *Itga1* knockdown predominantly modulated neurodegenerative processes and inflammatory pathways, consistent with our previous findings. Thus, comprehensive analyses of morphological alterations, polarization states, phagocytic capacity, and sequencing‐derived signaling pathways collectively demonstrated that *Itga1* knockdown effectively attenuated microglial hyperactivation.

To elucidate the downstream effectors of CD49a signaling, we conducted an in‐depth RNA‐Seq analysis, which identified *Pgam5* as a central node within inflammation‐associated pathways using KEGG network mapping. PGAM5 is predominantly located in mitochondria and is pivotal for mitochondrial homeostasis and inflammation [43, 59]. Alcoholic cardiomyopathy‐induced PGAM5 upregulation resulted in PGAM5‐dependent Phb2S91 dephosphorylation, leading to cardiac mitochondrial quality control (MQC) destabilization and mitochondrial dysfunction [60]. Enhanced PGAM5‐mediated Bax dephosphorylation and mitochondrial translocation was implicated in the development of acute kidney injury by initiating mitochondrial cytochrome c (Cyt c) release and activating the mitochondria‐mediated apoptotic pathway [61]. PGAM5 disrupts mitochondrial dynamics by promoting dynamin‐related protein 1 (DRP1) dephosphorylation, downregulating mitofusin‐2 (MFN2) and further enhancing doxorubicin‐induced oxidative stress and apoptosis [62]. PGAM5 depletion enhanced cell viability and reinstated mitochondrial dynamics, mitophagy, mitochondrial biogenesis, and mitochondrial unfolded protein response [45, 60, 63]. Our results demonstrated that microglial *Itga1* knockdown significantly downregulated PGAM5 expression in PD models and restored mitochondrial ultrastructure and physiological function. In contrast, OE‐PGAM5 abolished these protective effects. Mitochondrial homeostasis is intrinsically linked to inflammatory responses, with proinflammatory microglia consistently exhibiting impaired oxidative phosphorylation (OXPHOS) and mitochondrial dysfunction [64, 65, 66]. These findings collectively established PGAM5 as a critical downstream effector of CD49a, which exacerbated neuroinflammatory progression by promoting mitochondrial damage and disrupting homeostasis.

Persistent inflammation, which can be sustained by the NLRP3 inflammasome, the primary innate immune sensor for threatening signals, has been observed in various neurodegenerative diseases [67, 68]. Activation of the inflammasome sensor NLRP3 recruits ASC and effector caspase‐1, instigating significant tissue damage directly via pyroptosis and indirectly via IL‐18 and IL‐1β release [69]. We showed that microglial *Itga1* knockdown reduced NLRP3 and ASC expression and IL‐1β and IL‐18 secretion. However, these effects were reversed by OE‐PGAM5. Numerous studies have demonstrated that PGAM5 activation is intricately linked to the inflammatory response and NLRP3 inflammasome activation [46, 47, 70, 71]. After a spinal cord injury, eliminating PGAM5 may enhance mitochondrial function and diminish microglial activation and proinflammatory cytokine levels by elevating nuclear Nrf2 expression [70]. Traumatic brain injury (TBI) induced neuroinflammation may involve pathological coupling between PGAM5 and NLRP3 inflammasome assembly, with ASC oligomerization triggering IL‐1β‐mediated neurotoxicity via caspase‐1 activation [71]. Mechanistically, disrupting the PGAM5‐NEK7 connection impedes NLRP3 inflammasome activation in macrophages and alleviates DSS‐induced colitis in murine models [46]. In myocardial ischemia‐reperfusion injury, PGAM5‐MAVS condensates facilitate the activation of the NLRP3 inflammasome, likely functioning as a PGAM5/MAVS/NLRP3 sponge and enhancing caspase‐1‐mediated cleavage of GSDMD and IL‐18 [47].

There have been no reports on the relationship between PGAM5 and integrin receptors. One focus area of our future research would be to identify CD49a‐interacting proteins in proinflammatory microglia. Here, we explored the possible mechanisms via which CD49a affects PGAM5. Generally, integrin receptor activation facilitates the assembly of crucial adaptors, cytoskeletal components, and kinases at the cell membrane to form adhesion complexes that transduce signals from the extracellular matrix (ECM) to the cellular interior [18]. Irisin has been shown to alleviate mitochondrial damage and dyskinesia in PD models by upregulating the downstream AKT and ERK1/2 signaling pathways via integrin receptors rather than by directly targeting mitochondria [72]. Integrin and focal adhesion can regulate cytoskeleton distribution to govern actin‐related force remodeling. CD4^+^ T cells that largely lack integrin α1 expression have displayed reduced cell spreading and fibrillar actin [73]. The actin cytoskeleton regulator (eps‐8) modifies integrin signaling to promote mitochondrial homeostasis via cytoskeletal remodeling [74]. Ultraviolet B light induces integrin and F‐actin depletion, concomitant with impaired mitochondrial formation in the immortalized human keratinocyte HaCaT cell line [75]. Additionally, collagen I activates cell surface receptor integrins, promotes F‐actin polymerization, and ultimately enhances mitophagy [75]. The cytoskeleton can react to external mechanical stimuli via actin remodeling, affecting vital processes, including glycolysis, mitochondrial biogenesis, fission, and unfolded protein response. Mitochondrial fission transpires at mitochondria‐endoplasmic reticulum (ER) interface points and requires the assembly of actin filaments, which enable mitochondrial constriction and recruitment of the fission protein DRP1 [76, 77]. These results lead us to reasonably speculate that CD49a may affect actin and mitochondrial function, and PGAM5, as a key mitochondrial protein, plays a significant role in this process. Using phalloidin staining, we have proven that *Itga1* knockdown influences cytoskeletal rearrangement. However, the relationship among CD49a, actin, and PGAM5 requires further experimental verification.

We demonstrated that *Itga1* knockdown reduced the high reactivity of microglia and played a key role by inhibiting the downstream PGAM5‐ and NLRP3‐related signaling pathways. Although *Itga1* knockdown has shown therapeutic potential in animal experiments, it is not the most promising method in terms of clinical translation, considering its off‐target effects, long‐term safety concerns, and risk of drug resistance. Therefore, drugs that specifically block CD49a should be identified. Both jerdostatin and obtustatin are regarded as excellent integrin α1β1‐specific inhibitors and contain the key RTS or KTS tripeptide motifs [50, 78]. Studies have shown that KTS (such as obtustatin, viperistatin, and lebestatin) and RTS (Arg‐Thr‐Ser) (jerdostatin) disintegrins block integrin α1β1 more specifically than RGD (Arg‐Gly‐Asp)‐ disintegrins [79, 80, 81]. Among them, obtustatin attracts our attention due to its high activity (90 times stronger than jerdostatin), mature production system, and verified in vivo drug efficacy [82, 83]. Whereas the RTS motif demonstrates greater potency than KTS in inhibiting integrin α1β1, jerdostatin has lower activity than obtustatin, strongly indicating that modifications beyond the integrin‐binding motif account for the reduced inhibitory efficacy [78]. We performed molecular docking and MD to computationally validate specific intermolecular interactions between obtustatin and CD49a. Obtustatin effectively inhibited microglial adhesion to collagen IV and competitively blocked CD49a antibody binding to the CD49a ligand pocket. Preclinical and clinical evidences have demonstrated that obtustatin exhibits multifaceted therapeutic effects, including potent anti‐angiogenic activity, inhibition of tumor proliferation and invasion, and significant anti‐inflammatory properties [84, 85, 86, 87]. As a mature commercialized inhibitor, obtustatin is a prospective candidate for sarcoma therapy due to inhibition of angiogenesis at non‐toxic doses [84, 85]. Obtustatin ameliorates the hepatic tumor microenvironment, thereby suppressing cancer cell invasion through decellularized ECM derived from paclitaxel‐pretreated livers [86]. Besides, obtustatin effectively inhibits CD49a‐mediated eosinophil interactions with collagen IV and may emerge as a promising therapeutic agent for chronic inflammatory lung disorders [87]. Following intranasal administration of obtustatin in a PD mouse model, we documented significant amelioration of motor deficits, DA loss, and neuroinflammatory responses. These findings demonstrate the potent therapeutic efficacy of obtustatin against neuroinflammation and neurodegeneration, establishing its clinical translatability for PD intervention.

However, our study has several limitations. First, we identified CD49a as a pivotal mediator of chronic microglial hyperactivation. Beyond PD models, this mechanism warrants investigation in other neuroinflammatory diseases where CD49a^+^ microglial responses may similarly drive pathogenesis, including multiple sclerosis, stroke, and AD. Besides, we employed well‐established LPS‐ and MPTP‐induced PD models, which reliably recapitulate microglial activation and neuroinflammatory cascades. Since Lewy pathology represents a pathognomonic PD feature [88], future studies should evaluate CD49a‐targeted interventions in transgenic models exhibiting α‐synuclein aggregation. Validation in higher order systems, including porcine and non‐human primate PD models or patient‐derived cells, will provide critical preclinical validation of therapeutic applicability across PD subtypes. Second, our study delineated the causal hierarchy between CD49a and PGAM5 pathways, though the precise mechanism via which CD49a regulates PGAM5, mitochondrial homeostasis, and NLRP3 activation remains incompletely resolved. Identifying the direct molecular target of CD49a in microglia would elucidate its effector mechanisms and enable rational design of precise therapeutic interventions. Finally, while obtustatin demonstrates target‐specific CD49a binding and robust therapeutic efficacy in preclinical models, optimization of its administration regimen, alongside a comprehensive evaluation of its pharmacodynamics, safety profiles, and pharmacokinetics, remains imperative to de‐risk its clinical translation.

## 4 Conclusion

**FIGURE 11:**
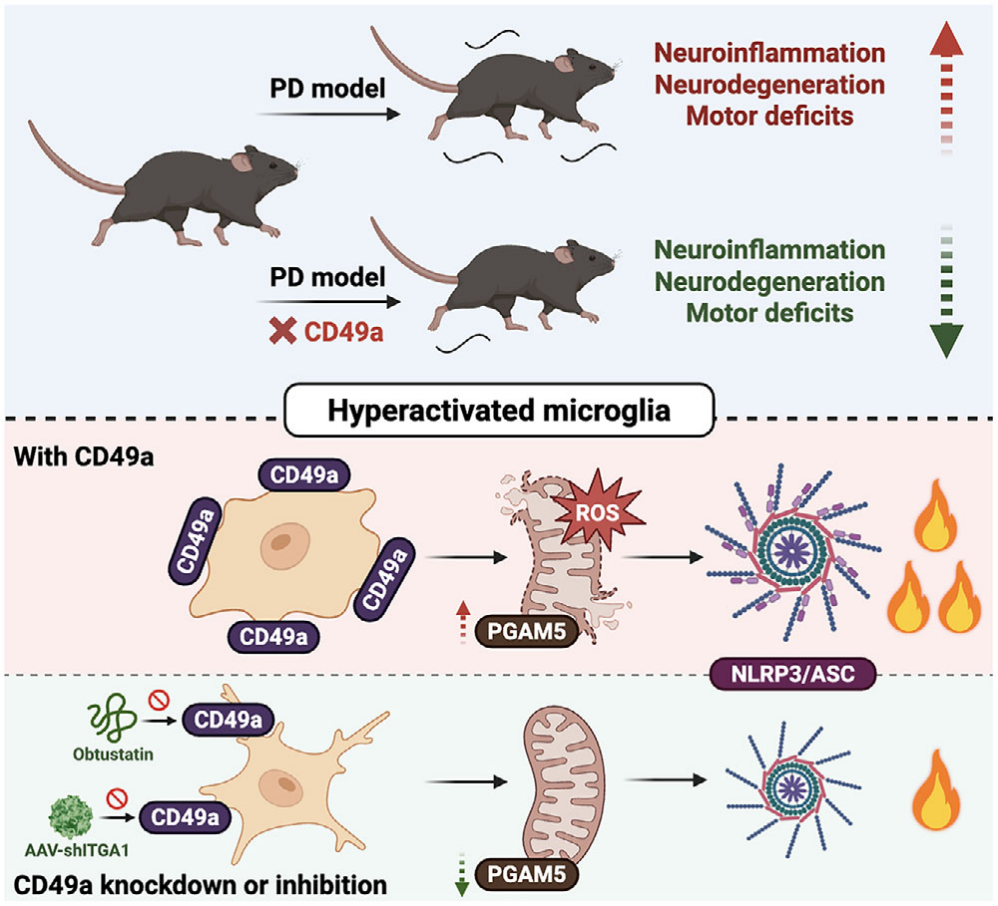
A schematic diagram shows the impact of CD49a in hyperactivated microglia on Parkinson's disease (PD) pathogenesis. Hyperactivated microglia drove chronic neuroinflammation and dopaminergic (DA) neurodegeneration via PGAM5‐mediated mitochondrial dysfunction and NLRP3 activation. Microglia‐specific knockdown of *Itga1* or CD49a‐targeted therapy with the selective disintegrin peptide obtustatin ameliorates motor deficits, underscoring its therapeutic potential.

In summary, our study delineated CD49a expression dynamics and its central regulatory role in chronically hyperactivated microglia during neuroinflammatory pathogenesis, with specific validation in PD models (Figure 11). CD49a exhibited predominantly microglial expression within the neural immune compartment, with significant upregulation particularly in hyperactivated microglia during chronic neuroinflammation. Specific *Itga1* knockdown attenuated microglial hyperreactivity and significantly improved motor deficits in PD models. Mechanistically, conditional microglial *Itga1* knockdown ameliorated mitochondrial dysfunction and suppressed NLRP3 inflammasome assembly via PGAM5 downregulation, thereby preserving DA neurons from neuroinflammatory degeneration. The integrin α1β1‐specific inhibitor obtustatin suppressed pathogenic microglial priming and preserved DA neuronal survival by specifically antagonizing CD49a conformational activation. The current study established a novel therapeutic strategy and identified obtustatin as a promising clinical candidate for PD treatment, with demonstrable translational potential for other neuroinflammatory and neurodegenerative pathologies.

## 5 Experimental Section

### 5.1 Reagents

LPS (L8274) and MPTP (M0896) were purchased from Sigma‐Aldrich (Burlington, USA). Nigericin (HY‐100381), obtustatin (HY‐P1408A), and dimethyl sulfoxide (DMSO) (HY‐Y0320) were obtained from MedChemExpress (New Jersey, USA). IL‐4 (214‐14‐20UG) and IFN‐γ (315‐05‐100UG) were acquired from PeproTech (Rocky Hill, USA). Anti‐ITGA1 (sc‐271034) and anti‐TH (sc‐25269) primary antibodies were obtained from Santa Cruz Biotechnology (Dallas, USA). An anti‐IBA1 primary antibody (ab178846) was acquired from Abcam plc (Cambridge, UK). Anti‐iNOS (18985‐1‐AP), anti‐COX2 (12375‐1‐AP), anti‐PGAM5 (28445‐1‐AP), anti‐NLRP3 (30109‐1‐AP), and anti‐ASC (83858‐3‐RR) primary antibodies were purchased from Proteintech (Wuhan, China). anti‐Actin (AA128) primary antibody, horseradish peroxidase (HRP)‐labeled goat anti‐rabbit IgG (H+L) (A0208), HRP‐labeled goat anti‐mouse IgG (H+L) (A0216) secondary antibodies, and prestained color protein marker (P0068; P0071) were acquired from Beyotime Biotechnology (Shanghai, China). Goat Anti‐mouse IgG (H+L) (DyLight 647 conjugate) (BA1151), Goat anti‐mouse IgG (H+L) (DyLight 550 conjugate) (BA1133), and Goat anti‐rabbit IgG (H+L) (DyLight 550 conjugate) (BA1135) secondary antibodies were obtained from Boster Biotechnology (Wuhan, China). APC anti‐mouse CD49a (142605), FITC anti‐mouse CD86 (105005), and PE anti‐mouse CD206 (141705) fluorochrome‐conjugated antibodies were purchased from BioLegend (San Diego, USA). Mouse IL‐1β (EK0394), IL‐6 (EK0411), IL‐18 (EK0433), and TNF‐α (EK0527) Enzyme‐Linked Immunosorbent Assay (ELISA) Kits were acquired from Boster Biotechnology.

### 5.2 Animals and Ethical Statement

Male mice (C57BL/6J background; 8–10 weeks old) were obtained from BesTest Bio‐Tech Co., Ltd. (Zhuhai, China). CX3CR1^Cre^ male mice (#025524; 8–10 weeks old) were obtained from Jackson Laboratory (Maine, USA). Animals were housed at the experimental animal center of Zhujiang Hospital at 25 ± 2°C and 55% ± 10% humidity with ad libitum access to food and water and a 12‐h light/12‐h dark cycle. Mice were allowed to acclimate to their new surroundings for a minimum of 1 week prior to initiating experiments. The Animal Care and Use Ethics Committee at Zhujiang Hospital approved all animal procedures (LAEC‐2023‐238).

### 5.3 PD Mouse Model Induction and Drug Treatment

An MPTP‐induced PD mouse model was established as described previously [89]. Specifically, MPTP was dissolved in saline to its working concentration 1 h pre‐injection. To establish a chronic PD model, mice received i.p. MPTP injections twice weekly at a dose of 25 mg/kg for 5 weeks. Equivalent volumes of saline were injected i.p. into control group mice. Obtustatin was initially dissolved in DMSO at a concentration of 25 mg/mL and was diluted to its working concentration. Healthy adult mice received i.n. (nasal drops) treatment of obtustatin (1, 2.5, and 5 mg/kg in 10 µL) 3 times per week for 5 weeks to assess its systemic tolerability and safety. In brief, serums and organs were harvested for biochemical and histological assessments of hepatorenal and immunological toxicity. Concurrently with chronic MPTP administration, mice were treated with i.n. obtustatin (1, 2.5, and 5 mg/kg in 10 µL) to determine its optimal therapeutic concentration.

### 5.4 Stereotaxic Surgery

Stereotaxic surgery was conducted in a stereotaxic apparatus (RWD Life Science Inc) under 1.25% tribromoethyl alcohol anesthesia. LPS experiment: The LPS model resembled a previously described one [90]. Specifically, 2 µg/µL LPS saline solution was prepared, and bilateral LPS microinjections of 500 nL were administered into the SNc of mice employing the following coordinates in relation to the bregma: A/P −3.3 mm, R/L ±1.3 mm, and D/V −4.5 mm. Control mice received equivalent volumes of saline injections. To evaluate CD49a expression in the LPS‐induced PD model, mice were split into Control and LPS groups. AAV experiment: AAV vectors for *Itga1* knockdown (AAV2/MG1.2‐CX3CR1‐lox2272‐loxP511‐shRNA(ITGA1)‐EGFP‐lox2272‐loxP511‐WPREs, AAV‐shITGA1) or empty vectors (AAV2/MG1.2‐CX3CR1‐lox2272‐loxP511‐shRNA(Control)‐EGFP‐lox2272‐loxP511‐WPREs, AAV‐shCon) were injected into the SNc of CX3CR1^Cre^ mice, as reported previously [37]. Each mouse was microinjected with either AAV‐shITGA1 or AAV‐shCon (100 nL of 5 × 10^12^ viral genome/µL) (BrainVTA Technology, Wuhan, China) using the above coordinates relative to the bregma. Mice were divided into shCon/saline, shITGA1/saline, shCon/MPTP, and shITGA1/MPTP groups.

### 5.5 Behavioral Tests

All tests were performed between 9:00 and 14:00 h and were assessed by the same rater in an observation room with low‐intensity light. The mice were acclimated for at least 1 h prior to starting the tests. To eliminate olfactory cues, equipment was cleaned with 75% ethanol between animals.

### 5.6 OFT

An OFT was performed to gauge the animals' locomotion and anxiety levels. EthoVision XT software (Beijing, China) was used to videotape mice for 10 min, while they were separately positioned at the middle of a box's bottom (50 × 50 × 40 cm). The entire traveled distance, distance in the center, and not moving time were noted.

### 5.7 Grip Strength Test

A grip strength test was used to assess the neuromuscular function of the upper extremities. The grip strength meter (76‐1066, Harvard Apparatus) was oriented horizontally, with mice being held by the tail and lowered toward the apparatus. Mice were permitted to grasp the bar and subsequently pulled backwards in the horizontal plane. The force exerted on the bar immediately prior to grip loss was documented as peak tension and quantified in Newtons.

### 5.8 Rotarod Test

Anti‐fatigue ability was assessed using an accelerated rotarod test. For 3 days, mice were trained twice daily on the TSE Rotarod (TSE Systems, Bad Homburg, Germany) at a speed of 5 rpm. In formal studies, mice were placed on a spinning rod that was progressively accelerated (0 to 30 rpm, 5 min) to measure fall duration.

### 5.9 Pole Test

Motor coordination was assessed using the pole test. Mice were positioned atop a vertical pole measuring 55 cm in length and 1 cm in diameter, with their heads oriented downwards. Mice underwent three days of training before the test. The total duration to reach the floor was recorded.

### 5.10 Microglial Isolation from Mouse Brain

Microglial isolation from mouse brains was conducted as described previously with certain modifications [64]. Briefly, mice aged 18–20 weeks were given an i.p. injection of pentobarbital sodium (40 mg/kg) to induce anesthesia, and saline was then infused intracardially. Using a Dounce homogenizer, newly obtained SN tissue was gently homogenized after being sliced into tiny pieces and suspended in Dounce buffer, which is 1.5 mM N‐2‐Hydroxyethylpiperazine‐N‐2‐Ethane Sulfonic Acid (HEPES) and 0.5% glucose in Hanks' Balanced Salt Solution (HBSS) buffer. To obtain cell pellets, brain tissue homogenates were suspended in phosphate buffered saline (PBS), filtered using 70 µm cell strainers, and centrifuged at 600 g for 6 min at 4°C. Myelin proteins were extracted through differential centrifugation using Percoll to achieve a single cell suspension. Erythrocytes were lysed to facilitate hemoglobin removal, while microglia were isolated employing CD11b^+^ MicroBeads (Miltenyi Biotec, 130‐093‐634).

### 5.11 RNA‐Seq and Bioinformatics Analysis

Microglial transcriptomics analysis was performed on samples from the shCon/Saline, shCon/MPTP, and shITGA1/MPTP groups. After combining isolated microglia from each of the five animals into a single sample, RNA extraction and purification were carried out (using AG RNAex Pro Reagent, AG21102). The library construction and sequencing processes were performed by BGI as previously reported. Briefly, 200–500‐bp library products were enriched, quantified, and ultimately paired‐end 150 bp (PE150) sequenced on a DNBSEQ‐T7 sequencer (MGI Tech Co., Ltd. China). The BGI Dr. Tom operating system, an in‐house customized BGI data mining system (http://biosys.bgi.com), was used to perform bioinformatics analysis, such as DEGs enrichment analysis, GO enrichment analysis, and KEGG pathway analysis. The raw RNA‐Seq data have been deposited in the Genome Sequence Archive (GSA) with the accession number (CRA034360) and are included in the Supporting Information.

### 5.12 Microglial Skeleton Analysis

Microglial skeleton analysis was carried out as previously reported [54, 91]. Briefly, a Z‐series stack of images was obtained with a confocal laser scanning microscope (CLSM) (AX/AX R, Nikon, Japan), and microglial skeleton analysis was conducted using ImageJ. After outlining and skeletonization, the soma area, number of endpoints, number of branches, and maximal branch length were determined using the AnalyzeSkeleton (2D/3D) plugin.

### 5.13 Cell Culture and Drug Treatment

The murine microglial BV2 cell line (CL‐0493; RRID: CVCL_0182) was obtained from Procell (Wuhan, China). BV2 cells were incubated in Dulbecco's Modified Eagle Medium/Nutrient Mixture F‐12 (DMEM/F12) (C11330500BT, Gibco, USA) with 10% heat‐inactivated fetal bovine serum (FBS) (a5669801, Gibco) and 1% penicillin‐streptomycin‐amphotericin B solution (100×) (P7630, Solarbio) in an incubator at 37°C and 5% CO_2_. Mouse DA neuronal MN9D cell line (SCC281; RRID: CVCL_M067) was obtained from Sigma‐Aldrich. MN9D cells were maintained in DMEM with 10% FBS and 1% penicillin‐streptomycin‐amphotericin B solution (100×) in an incubator at 37°C and 5% CO_2_. The cell lines used were confirmed to be contamination free. Primary neurons were isolated from the cortex of C57BL/6J mouse embryos at embryonic day 17, and primary cultured microglia were recovered from 3‐day‐old C57BL/6J mice. Primary neurons and microglia were acquired and cultivated as described previously [92, 93]. LPS/IFN‐γ stimulation, with standardized exposure to LPS (500 ng/mL) and IFN‐γ (20 ng/mL) for 24 h prior to functional assays, primed microglia towards a proinflammatory phenotype. Separately, a sequential NLRP3‐activating regimen involved 24‐h LPS priming, followed by a 45‐min nigericin (2.5 µm) challenge to trigger inflammasome assembly before downstream analyses.

### 5.14 RNA Interference

ITGA1 siRNAs, with specified sequences si‐NC: 5′‐UUCUCCGAACGUGUCACGUTT‐3′, si‐ITGA1‐1^#^: 5′‐GUUGGGAGAGAGAGAUCAAUGTT‐3′, and si‐ITGA1‐2^#^: 5′‐GGUUGGAAUUGUACAAUAUGGTT‐3′, were acquired from OBiO Biotech (Shanghai, China). As directed by the manufacturer, siRNAs were transfected into BV2 cells and primary microglia using Lipofectamine RNAiMAX Transfection Reagent (13778030, Invitrogen). Specifically, we transfected BV2 cells with 50 nM si‐ITGA1‐1^#^ or 50 nM si‐ITGA1‐2^#^ for 6 h, whereas a mixture of 20 nM si‐ITGA1‐1^#^ and 20 nM si‐ITGA1‐2^#^ (called si‐ITGA1‐mix) was used in primary microglia. After this, the medium was replaced, and cells were cultured for an additional 48 h before assessment of knockdown efficiency and subsequent experimentation.

### 5.15 Generation of PGAM5 Overexpression Cell Lines

The lentiviral transfer plasmid pSLenti‐CMV‐Pgam5‐3xFLAG‐PGK‐Puro‐WPRE or pSLenti‐CMV‐MCS‐3xFLAG‐PGK‐Puro‐WPRE (purchased from OBiO Biotech) was utilized to construct stable BV2 cell lines (OE‐PGAM5 or Vec‐PGAM5). Briefly, BV2 cells at 30% confluency were cultured in media containing appropriate dilutions of lentivirus (MOI = 10) along with polybrene (8 µg/mL). Following 48 h of transfection, the cells underwent puromycin selection (2.5 µg/mL) for 1 week to isolate the stably transfected cells.

### 5.16 Phagocytosis Assays

Phagocytosis assays were conducted as previously described [94]. Briefly, BV2 cells were plated into 24‐well plates and cultured overnight. After drug treatment, latex beads (aqueous suspension, 1.0 µm mean particle size) (L4655, Sigma) were dissolved with PBS at 1 µg/mL and incubated with cells for 30 min at 37°C. Microglial cells were washed three times with prewarmed PBS, fixed in 4% paraformaldehyde (PFA) (w/v) for 20 min, and permeabilized with Triton X‐100 (P0096, Beyotime) for another 20 min at room temperature. Next, cells were washed three times and incubated with Actin‐Tracker Red (C2205S, Beyotime) working solution for 30 min at room temperature. Subsequently, cells were sealed with 4',6‐diamidino‐2‐phenylindole (DAPI, ab104139, Abcam) for immunofluorescent testing. Images were acquired via a CLSM and analyzed using ImageJ software.

### 5.17 CM Assays

The CM assay methodology has been previously outlined [57]. Briefly, wild‐type BV2 cells or OE‐PGAM5 BV2 cells were pretreated with si‐NC or si‐ITGA1‐2^#^ by RNA interference, followed by treatment with PBS, LPS/IFN‐γ, or LPS/Nig. After two rounds of washing, the cells were cultivated for 24 h in fresh culture medium. Following treatments, CM from BV2 cells was collected and used to cultivate primary neurons or MN9D cells for 24 h in preparation for further tests.

### 5.18 Cell Viability Assay

Cell viability was assessed using a CCK8 assay (K1018, APExBIO Technology LLC, Houston, USA) in accordance with the manufacturer's guidelines. Briefly, MN9D cells were treated with CM for 24 h. Next, the cells were treated with a 10% CCK8 reagent for 1 h at 37°C The absorbance was measured at 450 nm by Multiscan Spectrum Microplate Reader (MSMR) (Synergy, BioTek, USA).

### 5.19 Cell Cytotoxicity Assay

Cell cytotoxicity was measured by a LDH Cytotoxicity Assay Kit (K2228, APExBIO). Briefly, MN9D cells were treated with CM for 24 h. A total of 50 µL of growth medium were collected and mixed with 50 µL of LDH working solution in 96‐well plates and incubated at room temperature for 30 min. Followed by 50 µL of stop solution, the absorbance was detected at 490 nm by MSMR to determine cell cytotoxicity.

### 5.20 PI Staining Assay

A PI staining assay (C1734, Beyotime) was carried out according to the manufacturer's instructions. Briefly, primary neurons were prepared into a single‐cell suspension after treated with CM for 24 h. Subsequently, the cells were centrifuged and, after supernatant removal, resuspended with a PI staining solution. Fluorescence intensities were measured using BD FACSVerse after incubation for 15 min at room temperature.

### 5.21 Detection of Mitochondrial Membrane Potential (Δψm)

Cells were washed with PBS three times before staining with a JC‐1 (C2003S, Beyotime) working solution. After using CLSM, the Δψm of labeled cells was calculated with ImageJ software. Low Δψm was reflected by green fluorescence (JC‐1 monomers), while high Δψm corresponded to red fluorescence (JC‐1 aggregates).

### 5.22 Detection of Intracellular ROS

An ROS assay kit (S0033, Beyotime) was used to detect intracellular ROS levels. Briefly, 20 µm dichloro‐dihydro‐fluorescein diacetate (DCFH‐DA) was added to BV2 cells after specific drug treatments. Next, the cells were incubated at 37°C for 20 min and washed with PBS three times, and fluorescence intensities were measured using BD FACSVerse.

### 5.23 Detection of Mitochondrial ROS

Mitochondrial ROS was detected with the Mitochondrial Superoxide Assay Kit (S0061S, Beyotime). Briefly, MitoSOX (a mitochondrial superoxide indicator) working solutions were added to BV2 cells after specific drug treatments. After incubation for 15 min at 37°C, the cells were washed with PBS three times, and fluorescence intensities were measured using BD FACSVerse.

### 5.24 TEM

TEM was employed to examine the ultrastructural morphology of mitochondria and synaptic vesicles in accordance with our earlier investigations [95, 96]. Briefly, various ethanol and acetone concentrations were used for tissue fixation and dehydration. Slices were then dyed with a 2% uranium acetate saturated alcohol solution and 2.6% lead citrate after tissues had been embedded with an 812 embedding agent (SPI‐Pon 812 Epoxy Resin Monomer; SPI, Shanxi, China). TEM (HT7700; Hitachi, Tokyo, Japan) was used for imaging and image analysis.

### 5.25 Flow Cytometry Analysis

After drug treatment, single‐cell suspensions of BV2 cells or primary microglia were prepared. The cells were centrifuged and, after supernatant removal, resuspended in Cell Staining Buffer (420201, BioLegend). Next, the cells were washed three times, blocked with TruStain FcX PLUS (anti‐mouse CD16/32) (156603, BioLegend), and stained with the following fluorochrome‐conjugated antibodies (all purchased from BioLegend) for 30 min at 4°C: APC anti‐mouse CD49a (142605), FITC anti‐mouse CD86 (105005), and PE anti‐mouse CD206 (141705). Data were acquired immediately by BD FACSVerse and analyzed using FlowJo software.

### 5.26 RNA Extraction and Quantitative Real‐Time Polymerase Chain Reaction (qRT‐PCR)

Total RNA was extracted from cells using the AG RNAex Pro Reagent (AG21102, Accurate Biotechnology, Changsha, Hunan) and reverse transcribed utilizing the Evo M‐MLV RT Mix Kit (AG11728, Accurate Biotechnology). Validated primers were purchased from BGI Genomics (Shenzhen, China). Then, qRT‐PCR was performed using SYBR Green Pro Taq HS SuperMix (AG11728, Accurate Biotechnology) on an ABI QuantStudio 3 Real‐Time PCR System (Life Technologies, Carlsbad, USA). *Actb* served as an internal reference gene. Data analysis was performed using the ΔΔCt method, unless otherwise stated. All qPCR primers were designed with PrimerBank (https://pga.mgh.harvard.edu/primerbank, Cambridge, USA). Detailed sequences are provided in Table S2 (Supporting Information).

### 5.27 Molecular Docking

GRAMM (http://gramm.compbio.ku.edu/) was employed for protein‐peptide molecular docking to investigate the interaction between integrin α1β1 and obtustatin [97]. GRAMM implements a grid‐based, rigid‐body docking algorithm optimized for predicting macromolecular interfaces. The workflow consisted of: (1) molecular preprocessing; (2) grid space generation; (3) rigid‐body docking via Fast Fourier Transform sampling; (4) pose selection and refinement. The top 10 complexes ranked by their shape complementarity score were retained, with the highest‐scoring conformation selected for structural analysis; and (5) binding interface visualization. Hydrogen bonding networks and hydrophobic interactions were visualized using PyMOL v2.6 [98].

### 5.28 Immunofluorescence and Immunohistochemistry Assays

For in vitro immunofluorescence experiments, BV2 cells, MN9D cells, primary microglia, or primary neurons were fixed in 4% PFA for 20 min at room temperature. Cells were then permeabilized with Triton X‐100 (P0096, Beyotime) or Saponin (P0095, Beyotime) for an additional 20 min. For in vivo immunofluorescence assays, mouse brain sections (7–30 µm thick) were prepared using a cryostat (CM1950, Leica). After that, the slices were subjected to fixation and permeabilization, followed by the same subsequent steps as described above. After being washed three times with PBS, cells were blocked with 2% bovine serum albumin (BSA, ST023, Beyotime) in PBS for 45 min at 37°C. Cells were then incubated overnight at 4°C with the following primary antibodies: anti‐ITGA1 (1:200), anti‐TH (1:500), and anti‐IBA1 (1:1000). After proper rinsing, cells were incubated with corresponding fluorescently labeled secondary antibodies for 45 min at 37°C. Subsequently, cells were sealed with DAPI for the immunofluorescent test. Images were scanned using a CLSM and analyzed using ImageJ software. For in vivo immunohistochemistry experiments, brain slices were exposed to secondary antibodies coupled with biotin for 45 min at 37°C and then stained with 3,3N‐diaminobenzidine tertrahydrochloride (DAB) substrate solution (P0203, Beyotime) at room temperature until optimal chromogenic signal intensity was achieved. Images were captured using Stereo microscopy (M165 FC, Leica, Germany) and analyzed by ImageJ software.

### 5.29 Western Blot (WB) Assay

Total proteins were extracted from BV2 cells, MN9D cells, primary microglia, primary neurons, or SN tissue using RIPA Lysis Buffer (P0013, Beyotime). Protein concentration was determined via the Bicinchoninic Acid (BCA) Protein Assay Kit (P0010, Beyotime). Western blotting was performed as previously described [99]. Briefly, approximately 20 µg of each protein sample was separated by sodium dodecyl sulfate polyacrylamide gel electrophoresis (SDS‐PAGE) and then transferred onto polyvinylidene difluoride (PVDF) membranes (Millipore, USA). The membranes were incubated with the indicated primary antibodies on the shaker at 4°C overnight: anti‐ITGA1, anti‐TH, anti‐iNOS, anti‐COX2, anti‐PGAM5, anti‐NLRP3, anti‐ASC, and anti‐Actin. Subsequently, HRP‐labeled secondary antibodies were incubated for 1 h on the shaker at room temperature. After being washed three times, the membranes were incubated with Immobilon Western Chemiluminescent HRP Substrate (WBKLS0100, Millipore) to visualize the bound antibodies. Chemiluminescent signals were detected using a UVITEC Q9 imaging system (Cambridge, UK), and band optical densities were quantified with ImageJ software. All experiments were performed in triplicate.

### 5.30 ELISA

Mouse striatal concentrations of IL‐1β, IL‐6, IL‐18, and TNF‐α were quantified using ELISA kits (Boster Biotechnology, China) following the details of a previous study [100]. Briefly, striatal samples were homogenized in ice‐cold PBS containing protease inhibitors. Then, homogenates were then centrifuged to collect supernatants, which were then incubated with antibody‐precoated plates and HRP‐conjugated secondary antibodies at 37°C for 1 h. After adding chromogenic reagents A and B and incubating at 37°C for 15 min, absorbance (OD) was measured at 450 nm using MSMR. Protein concentrations in supernatants were determined via BCA assay, and cytokine levels were expressed as pg/mL (normalized to total protein content).

### 5.31 Statistical Analysis

GraphPad Prism 10.0 (GraphPad Software Inc., La Jolla, CA, USA) was used for statistical analysis. Significant differences were evaluated using Student's *t*‐test (*t*‐test) or one‐way or two‐way analysis of variance (ANOVA) followed by Dunnett's or Tukey's multiple comparisons test. The *p*‐values are presented as ^*^ *p* < 0.05, ^**^ *p* < 0.01, ^***^ *p* < 0.001, and ^****^ *p* < 0.0001, whereas “ns” indicates “no significant difference”. Values are presented as mean ± standard error of the mean (SEM).

## Author Contributions

H.L., R.C., and Y.G. developed the concept of the study; H.L., R.C., and Y.G. designed the experiments; H.L., Y.Z., X.W., Z.W., and Z.X. performed the experiments; H.L., Y.Z., X.W., Z.W., and Z.X. collected and analyzed the data; H.L. and Y.Z. wrote the manuscript. H.L., R.C., and Y.G. supervised the study. All the authors read and approved the final manuscript.

## Funding

This work was supported by grants from National Natural Science Foundation of China (Nos. 82271488 and 82471468 to Y.G., 82271859 to R.C.) and Guangdong Natural Science Foundation (No. 2024A1515010512 to Y.G., 2023A1515010639 to R.C.).

## Ethical Approval

The study complies with institutional ethical guidelines for animal care and was approved by the responsible authorities at Zhujiang Hospital (LAEC‐2023‐238).

## Conflicts of Interest

The authors declare no conflict of interest.

## Data and Materials Availability

The data that support the findings of this study are available from the corresponding author upon reasonable request.

## Supporting information




**Supporting File 1**: advs73461‐sup‐0001‐SuppMat.docx.

**Supporting File 2**: advs73461‐sup‐0002‐DataFile.zip.
