# Supplementary material for: Targeting Microglial CD49a Inhibits Neuroinflammation and Demonstrates Therapeutic Potential for Parkinson's Disease
Source: Adv Sci (Weinh). 2025 Dec 29;13(13):e15138. doi: 10.1002/advs.202515138 (PMC12955899; doi:10.1002/advs.202515138)
Supplement: Supplementary file 1 — Supporting File 1: advs73461‐sup‐0001‐SuppMat.docx. [file ADVS-13-e15138-s002.docx]

**Supporting Information for**

**Targeting Microglial CD49a Inhibits Neuroinflammation and Demonstrates Therapeutic Potential for Parkinson's Disease**

*Huanpeng Lu,^*^ Yunmin Zhu, Xi Wang, Zelin Wu, Zijian Xu, Rongqing Chen,^*^ Yanwu Guo^*^*

Corresponding author: Huanpeng Lu, lhpv427@163.com; Rongqing Chen, creatego@hotmail.com; Yanwu Guo, eguoyanwu@163.com

**This file includes:**

Supplementary Materials and Methods

Figures S1 to S7

Tables S1 and S2

References (1 to 6)

**Supplementary Materials and Methods**

*Hoechst staining*: Hoechst 33258 (C1017, Beyotime Biotechnology, China) working solutions were added to BV2 cells (a murine microglial cell line) after specific drug treatment and incubated for 5 minutes at room temperature. After washed three times, fluorescence intensities were measured using BD FACSVerse (Becton, Dickinson and Company, USA) and analyzed using FlowJo software.

*Cell adhesion assay*: 96-well plates were coated with Collagen IV (356233, Corning Inc., USA) at a concentration of 1 µg/cm², dissolved in 0.05 M HCl, and incubated overnight at 4°C. Following three washes with Phosphate Buffered Saline (PBS), the wells were incubated in a blocking solution consisting of 0.1% bovine serum albumin (BSA, ST023, Beyotime Biotechnology, China) in PBS for 1 hour. 100 µL of primary microglia supernatant in Dulbecco's Modified Eagle Medium/Nutrient Mixture F-12 (DMEM/F12) (C11330500BT, Gibco, USA) without FBS and containing varying concentrations (1, 2.5, and 5 µM) of obtustatin (1:1) were then put into the wells and incubated at 37°C for 1 hour in an incubator at 37°C and 5% CO_2_. Following three washes with PBS, cells were fixed in 4% paraformaldehyde (PFA) (w/v) for 20 minutes and subsequently stained with Crystal Violet Staining Solution (C0121, Beyotime Biotechnology) for 10 minutes. Following three washes with PBS, images were scanned using a Stereo microscope (M165 FC, Leica, Germany) and analysed using ImageJ software.

*Antibody competition assay*: Obtustatin at the different concentrations (1, 2.5, and 5 µM) were mixed with APC anti-mouse CD49a (1:200) for 10 minutes. Next, the mixture was subsequently incubated with BV2 cells for an additional 10 minutes at 37°C. Data were collected promptly using BD FACSVerse and subsequently analyzed with FlowJo software.

*Molecular docking*: GRAMM (http://gramm.compbio.ku.edu/) was employed for protein-peptide molecular docking to investigate the interaction between integrin α1β1 and obtustatin. ^1^ GRAMM implements a grid-based rigid-body docking algorithm optimized for predicting macromolecular interfaces. The workflow consisted of: (1) molecular preprocessing; (2) grid space generation; (3) rigid-body docking via Fast Fourier Transform sampling; (4) pose selection and refinement. The top 10 complexes ranked by their shape complementarity score were retained, with the highest-scoring conformation selected for structural analysis; and (5) binding interface visualization. Hydrogen bonding networks and hydrophobic interactions were visualized using PyMOL v2.6. ^2^

*Molecular dynamics simulations (MD)*: This study conducted 100-ns MD using GROMACS v2022.03 on the protein complex modeled by AlphaFold^3^, employing the AMBER99SB-ILDN force field. ^4^ Simulation parameters followed established protocols. ^5^ For trajectory analysis, key parameters including root-mean-square deviation (RMSD), root-mean-square fluctuation (RMSF), radius of gyration (Rg), solvent-accessible surface area (SASA), and hydrogen bonding patterns were evaluated. Gibbs free energy landscapes were computed based on RMSD and Rg coordinates using the native "g_sham" module and "xpm2txt.py" conversion script within GROMACS v2022.03. The molecular mechanics/Poisson-Boltzmann surface area (MM/PBSA) method, implemented in MMPBSA.py v16.0, was employed to determine binding free energies. ^6^

*Statistical analysis*: GraphPad Prism 10.0 (GraphPad Software Inc., La Jolla, CA, USA) was used for statistical analysis. Significant differences were evaluated using Student's *t*-test (*t*-test) or one-way or two-way analysis of variance (ANOVA) followed by Dunnett's or Tukey's multiple comparisons test. The p-values are presented as **P*<0.05, ***P*<0.01, ****P*<0.001, and *****P*<0.0001, whereas ns indicates no significant difference. Values are presented as mean ± standard error of the mean (SEM).

**Supplementary Figures**


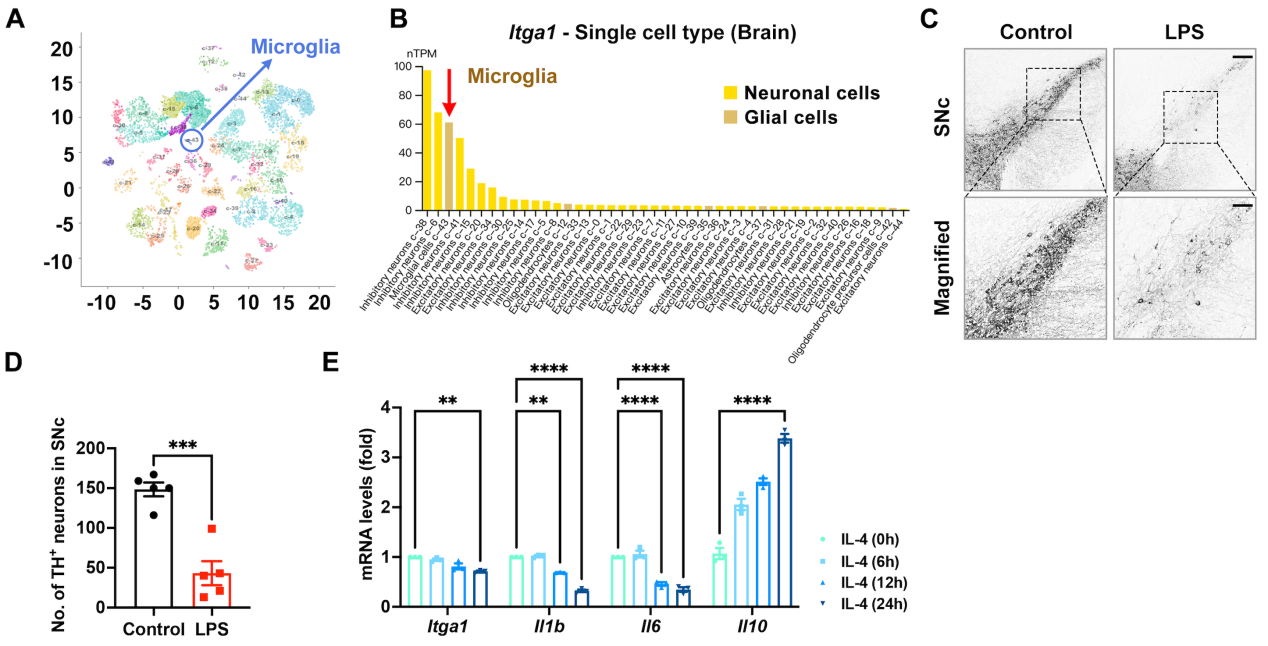


**Figure S1.** *Itga1* is predominantly expressed in microglia and downregulated under IL-4 stimulation. A,B) The Human Protein Atlas (HPA) and cell type-specific expression quantification of *Itga1*. The arrow indicates the transcript abundance of *Itga1* in microglia. C,D) Representative immunohistochemical staining images with quantitative analysis of tyrosine hydroxylase (TH)^+^ neurons in the substantia nigra pars compacta (SNc) (*n* = 5). Scale bar: 100 μm for original and 50 μm for magnified images in the SNc. E) The mRNA transcript abundance of *Itga1*, *Il1b*, *Il6*, and *Il10* in IL-4-treated primary microglia for distinct durations (0, 6, 12, and 24 hours; *n* = 3). The concentration of IL-4 is 20 ng/mL. Data are represented as means ± SEM with *t*-test and two-way ANOVA. **P*<0.05, ***P*<0.01, ****P*<0.001, and *****P*<0.0001.


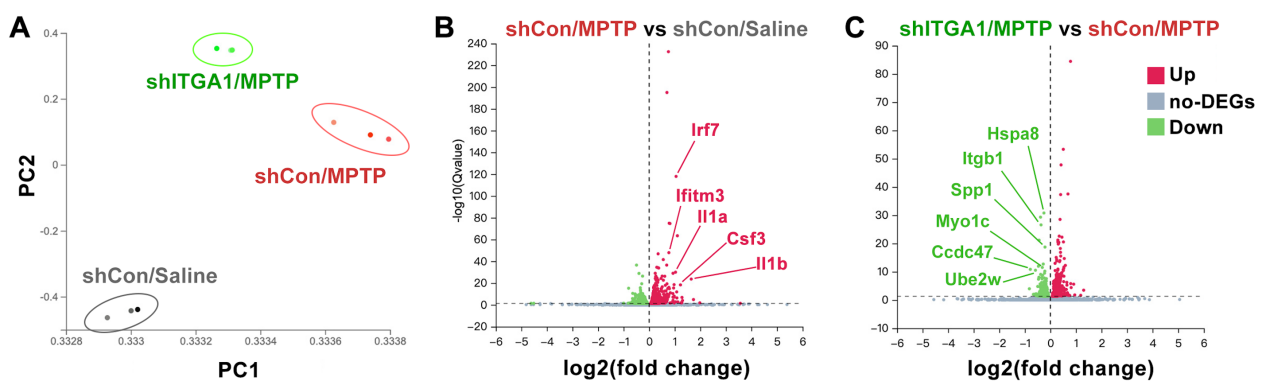


**Figure S2.** Microglial transcriptome analysis from three groups. A) Microglial transcriptomics samples among shCon/Saline, shCon/MPTP, and shITGA1/MPTP groups analyzed by principal component analysis (PCA) plots (*n* = 3). B,C) Volcano plots showing the differentially expressed genes (DEGs) between the "shCon/MPTP *vs* shCon/Saline" groups as well as the "shITGA1/MPTP *vs* shCon/MPTP" groups. Upregulated DEGs, red; downregulated DEGs, green; no DEGs, gray. Key DEGs in red (*Irf7*, *Ifitm3*, Csf3, *Il1a*, and *Il1b*) and green (*Hspa8*, *Itgb1*, *Spp1*, *Myo1c*, *Ccdc47*, and *Ube2w*) have been identified and labeled.


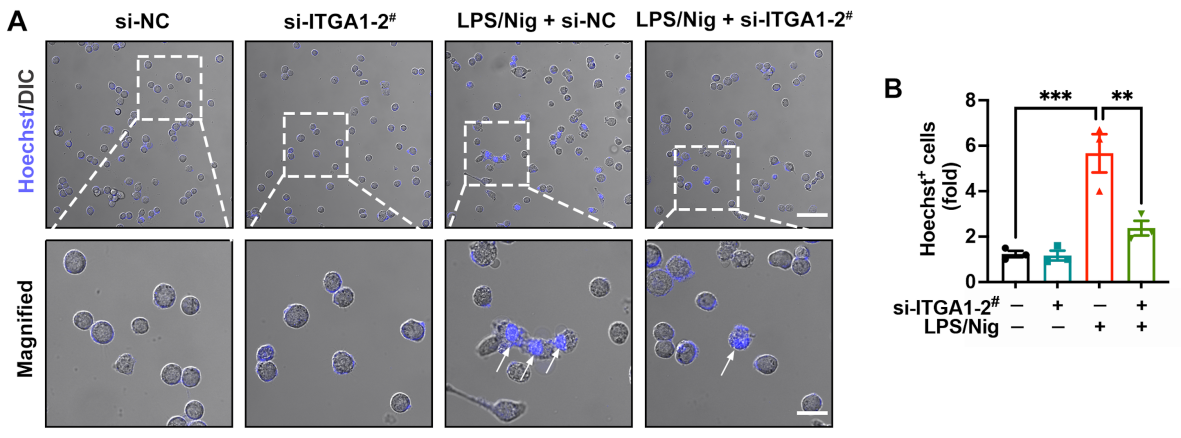


**Figure S3.** *In vitro* microglial *Itga1* knockdown alleviates mitochondrial damage-associated cell death. A,B) Representative digital image correlation (DIC) plus fluorescence images and quantification of Hoechst^+^ BV2 cells (*n* = 3). White arrows indicate Hoechst^+^ cells. Scale bar: 50 μm for original and 20 μm for magnified images. Data are represented as means ± SEM with ordinary one-way ANOVA. **P*<0.05, ***P*<0.01, ****P*<0.001, and *****P*<0.0001.


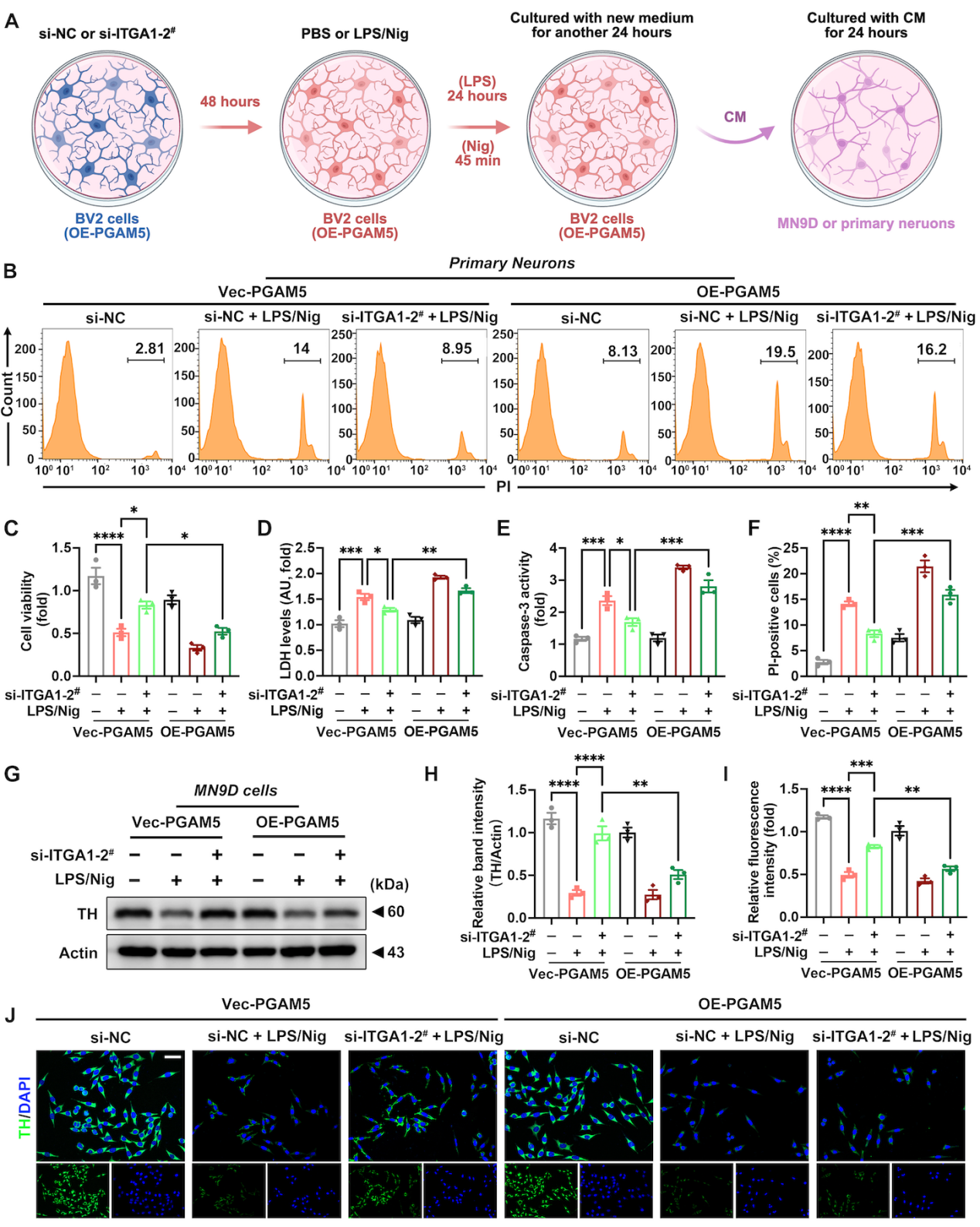


**Figure S4.** Microglial PGAM5 overexpression aggravates neuronal death. A) The schematic diagram of the microglial conditioned medium (CM) treatment on MN9D or primary neurons. MN9D or primary neurons were cultured with CM from PGAM5 overexpression (OE-PGAM5) or empty vector control (Vec-PGAM5) BV2 cells for 24 hours. B,F) Representative flow cytometry (FC) images and quantification of propidium iodide (PI) staining in primary neurons (*n* = 3). C,D) Quantification of Cell Counting Kit-8 (CCK8) assay and lactate dehydrogenase (LDH) assay in primary neurons (*n* = 3). E) Quantification of caspase-3 activity in primary neurons (*n* = 3). G,H) Representative blots and quantification of TH expression in MN9D cells (*n* = 3). I,J) Quantification and representative immunofluorescence images of TH expression in MN9D cells (*n* = 3). TH, green fluorescence; DAPI, blue fluorescence. Scale bar: 50 μm. Data are represented as means ± SEM with one-way ANOVA. **P*<0.05, ***P*<0.01, ****P*<0.001, and *****P*<0.0001.


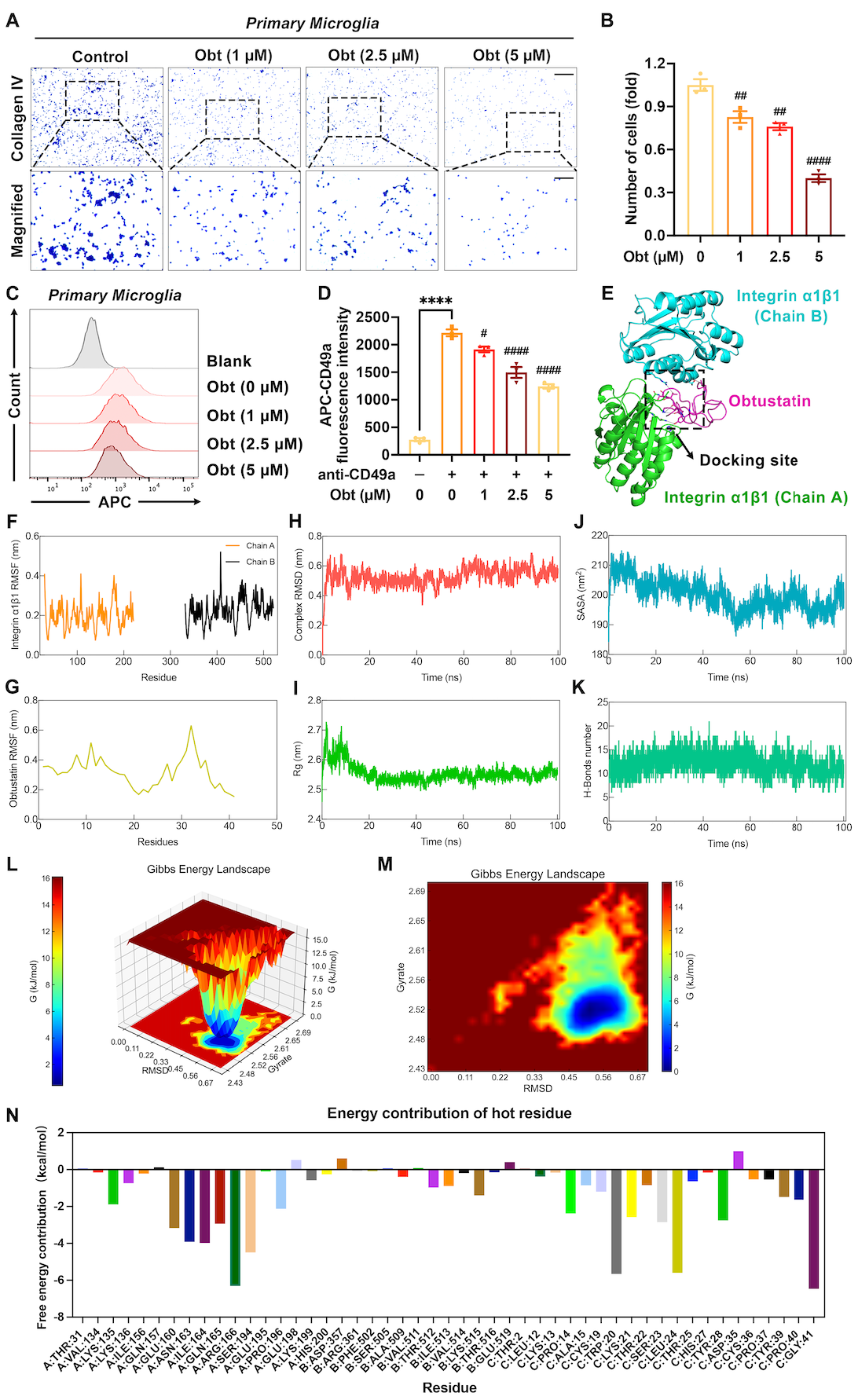


**Figure S5.** Obtustatin specifically binds to and blocks integrin α1β1. A,B) Representative images and quantification of cell adhesion assay by crystal violet staining (*n* = 3). The effect of obtustatin (0, 1, 2.5, and 5 µM) on primary microglia adhesion to collagen IV. Obt, Obtustatin. Scale bar: 500 µm for original and 200 µm for magnified images. C,D) Representative FC images of the antibody competent assay (APC anti-mouse CD49a and different concentrations of Obt) and quantification of APC-CD49a fluorescence intensity in primary microglia (*n* = 3). E) Cartoon representation of molecular docking of Obt (PDB: 1MPZ) with integrin α1β1 (PDB: 1QC5). Black arrow represents the docking site. F,G) RMSF profiles of integrin α1β1 and Obt. H–K) RMSD, Rg, SASA, and H-Bonds number profiles of the complex over 100 ns. L,M) Gibbs energy landscape of the complex. N) Energy contribution of hot residue. Data are represented as the mean ± SEM with one-way ANOVA. **P*<0.05, ***P*<0.01, ****P*<0.001, and *****P*<0.0001 *vs* the blank group. ^#^*P*<0.05, ^##^*P*<0.01, ^###^*P*<0.001, and ^####^*P*<0.0001 *vs* the control or anti-CD49a only group. ns, not significant.


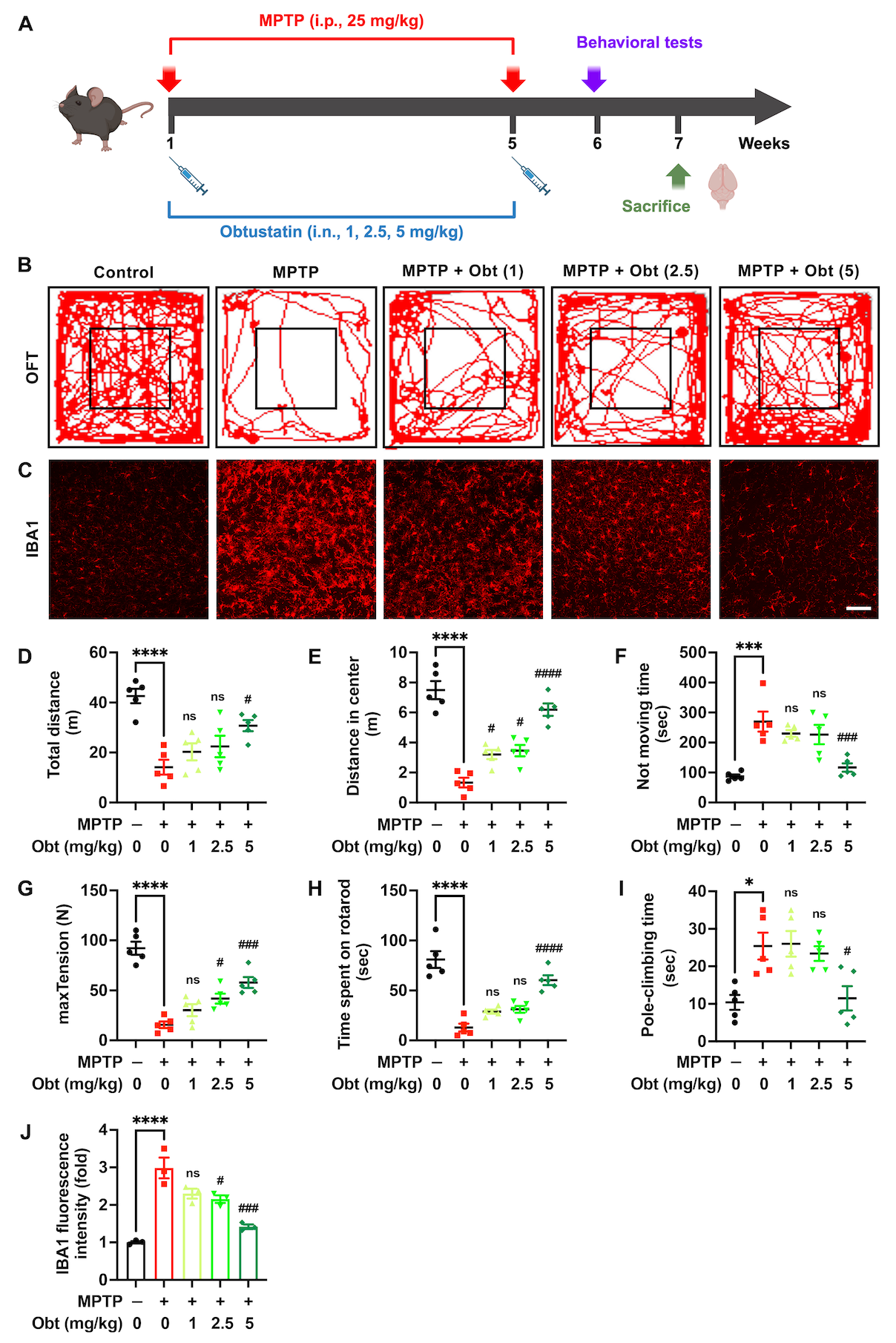


**Figure S6.** Optimal therapeutic concentration of obtustatin for animal experiments. A) Experimental design involving obtustatin treatment (intranasal (i.n.); 1, 2.5, and 5 mg/kg) in MPTP-induced PD mice (i.p.; 25 mg/kg). Obtustatin and MPTP were administered twice a week for 5 weeks. Obtustatin was administered in the morning and MPTP in the afternoon, with an interval of 6 hours. Behavioral tests in the 6^th^ week. Sacrifice in the 7^th^ week. B) Travelled trace of mice in open field test (OFT). C) Representative immunofluorescence staining of IBA1 in the SNc. IBA1, red fluorescence. Scale bar: 100 μm. D–I) Quantification of total distance, distance in center, and not moving time in OFT as well as quantification of grip strength test, rotarod test, and pole-climbing test (*n* = 10). J) Quantification of IBA1 positive density in the SNc (*n* = 3). Data are represented as the mean ± SEM with one-way ANOVA. **P*<0.05, ***P*<0.01, ****P*<0.001, and *****P*<0.0001 *vs* the control group. ^#^*P*<0.05, ^##^*P*<0.01, ^###^*P*<0.001, and ^####^*P*<0.0001 *vs* the MPTP group. ns, not significant.


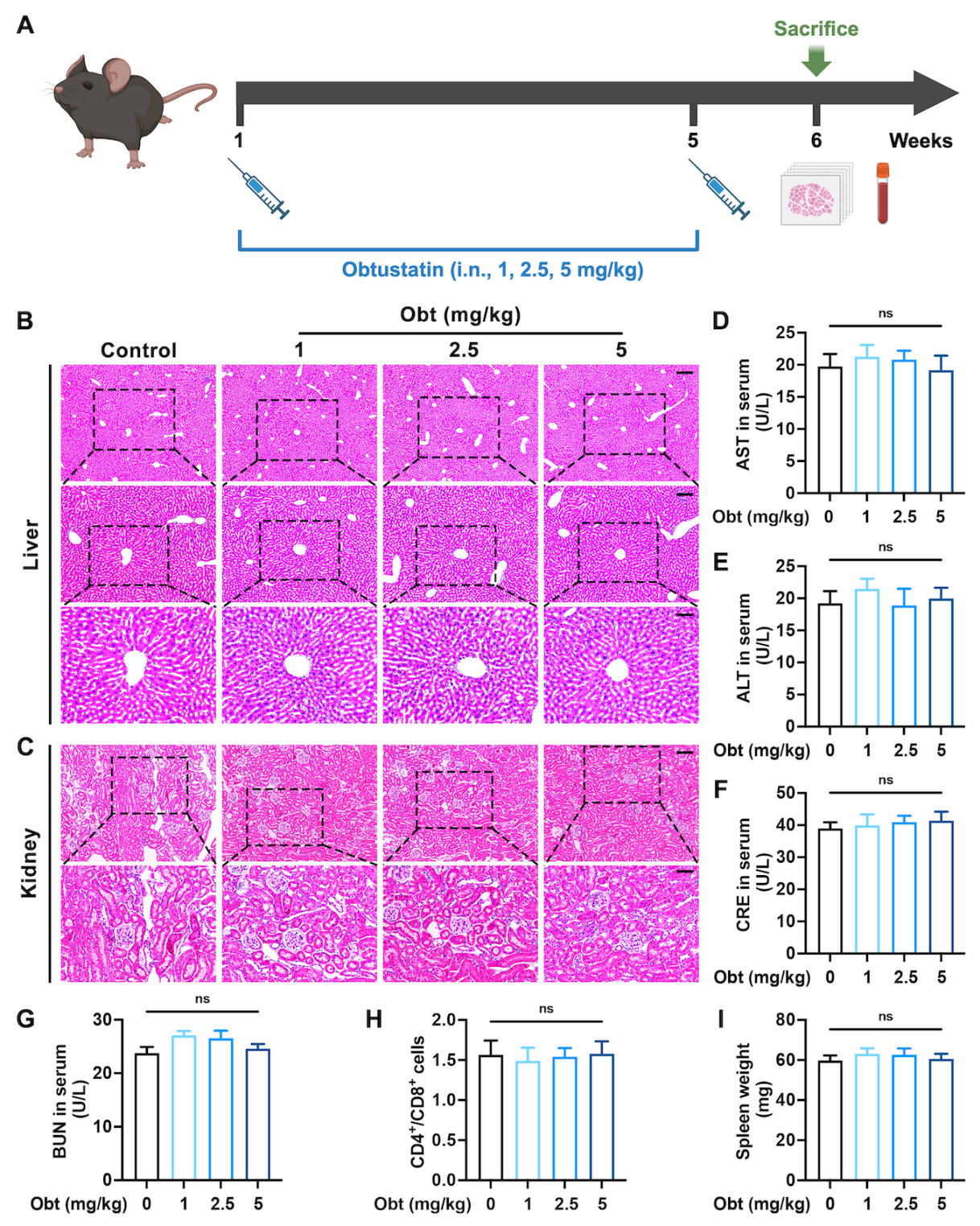


**Figure S7.** *In vivo* toxicity test of obtustatin. A) Experimental design involving obtustatin treatment (i.n.; 1, 2.5, and 5 mg/kg) in C57 mice. Obtustatin was administered twice a week for 5 weeks. Sacrifice in the 7^th^ week including the collection of liver, kidney, spleen, and blood. B) Representative images of hematoxylin-eosin (H&E) staining in the liver. Scale bar: 500 µm for the top, 200 µm for the middle, and 100 µm for the under images. C) Representative images of H&E staining in the kidney. Scale bar: 200 µm for the top and 100 µm for the under images. D–G) Quantification of aspartate aminotransferase (AST), alanine aminotransferase (ALT), creatinine (CRE), and blood urea nitrogen (BUN) levels in the serum (*n* = 5). H,I) Quantification of CD4^+^/CD8^+^ cells in the spleen and spleen weight (*n* = 5). Data are represented as the mean ± SEM with one-way ANOVA. ns, not significant.

**Supplementary Tables**

**Table S1. Oligonucleotide primers for target gene amplification.​**

| Primers | 5'-3' |
| --- | --- |
| *Itga1* | CGCTGTGAATCAGACGAGGT |
| *Il1b* | TGCCACCTTTTGACAGTGATG |
| *Il6* | TGGTCTTCTGGAGTACCATAGC |
| *Tnf* | GATCGGTCCCCAAAGGGATG |
| *Il10* | GCTGTCATCGATTTCTCCCCT |
| *Il13* | TCACACAAGACCAGACTCCCCT |
| *Arg1* | GTAGACCCTGGGGAACACTAT |
| *Actb* | CACTGTCGAGTCGCGTCC |

**Table S2. The average binding free energy (kcal/mol) of the complex was calculated by the MM/PBSA method.**

| Energy contributions | Complex |
| --- | --- |
| ΔE_vdW_ | -83.66 |
| ΔE_elec_ | -650.22 |
| ΔE_GB_ | -650.22 |
| ΔE_surf_ | -12.25 |
| ΔG_gas_ | -733.88 |
| ΔG_solvation_ | 671.81 |
| ΔG_total_ | -62.07 |

ΔE_vdW_, Change in van der Waals energy; Δ_Eelec_, Change in electrostatic energy; ΔE_GB_, Change in Generalized Born solvation energy; ΔE_surf_, Change in nonpolar solvation surface energy; ΔG_gas_, Change in gas-phase free energy; ΔG_solvation_, Change in solvation free energy; ΔG_total_, Change in total free energy. ΔG_total_ = ΔG_gas_ + ΔG_solvation_. ΔG_total_ is an important indicator reflecting the interaction affinity.

**References**

1. Singh A, Tytarenko AM, Ambati VK, et al. GRAMMCell: Docking-based Cell Modeling Resource. *J Mol Biol*. Aug 1 2025;437(15):169085. doi:10.1016/j.jmb.2025.169085

2. Lu XJ. DSSR-enabled innovative schematics of 3D nucleic acid structures with PyMOL. *Nucleic Acids Res*. Jul 27 2020;48(13):e74. doi:10.1093/nar/gkaa426

3. Oanca G, van der Ent F, Åqvist J. Efficient Empirical Valence Bond Simulations with GROMACS. *J Chem Theory Comput*. Sep 12 2023;19(17):6037-6045. doi:10.1021/acs.jctc.3c00714

4. Briand E, Kohnke B, Kutzner C, Grubmüller H. Constant pH Simulation with FMM Electrostatics in GROMACS. (A) Design and Applications. *J Chem Theory Comput*. Feb 25 2025;21(4):1762-1786. doi:10.1021/acs.jctc.4c01318

5. Li N, Yang MY, Zhang MY, et al. Exploring the distinct activation mechanisms of neuromedin B receptor through multiple replica molecular dynamics simulations and Markov state modeling. *Acta Pharmacol Sin*. Jun 27 2025;doi:10.1038/s41401-025-01603-w

6. Bettadj FZY, Benchouk W, Guendouzi A. Computational exploration of novel ketoprofen derivatives: Molecular dynamics simulations and MM-PBSA calculations for COX-2 inhibition as promising anti-inflammatory drugs. *Comput Biol Med*. Dec 2024;183:109203. doi:10.1016/j.compbiomed.2024.109203
