# Supplementary figures and images for: Targeting Microglial CD49a Inhibits Neuroinflammation and Demonstrates Therapeutic Potential for Parkinson's Disease
Source: Adv Sci (Weinh). 2025 Dec 29;13(13):e15138. doi: 10.1002/advs.202515138 (PMC12955899; doi:10.1002/advs.202515138)

**Uncropped Western blots**


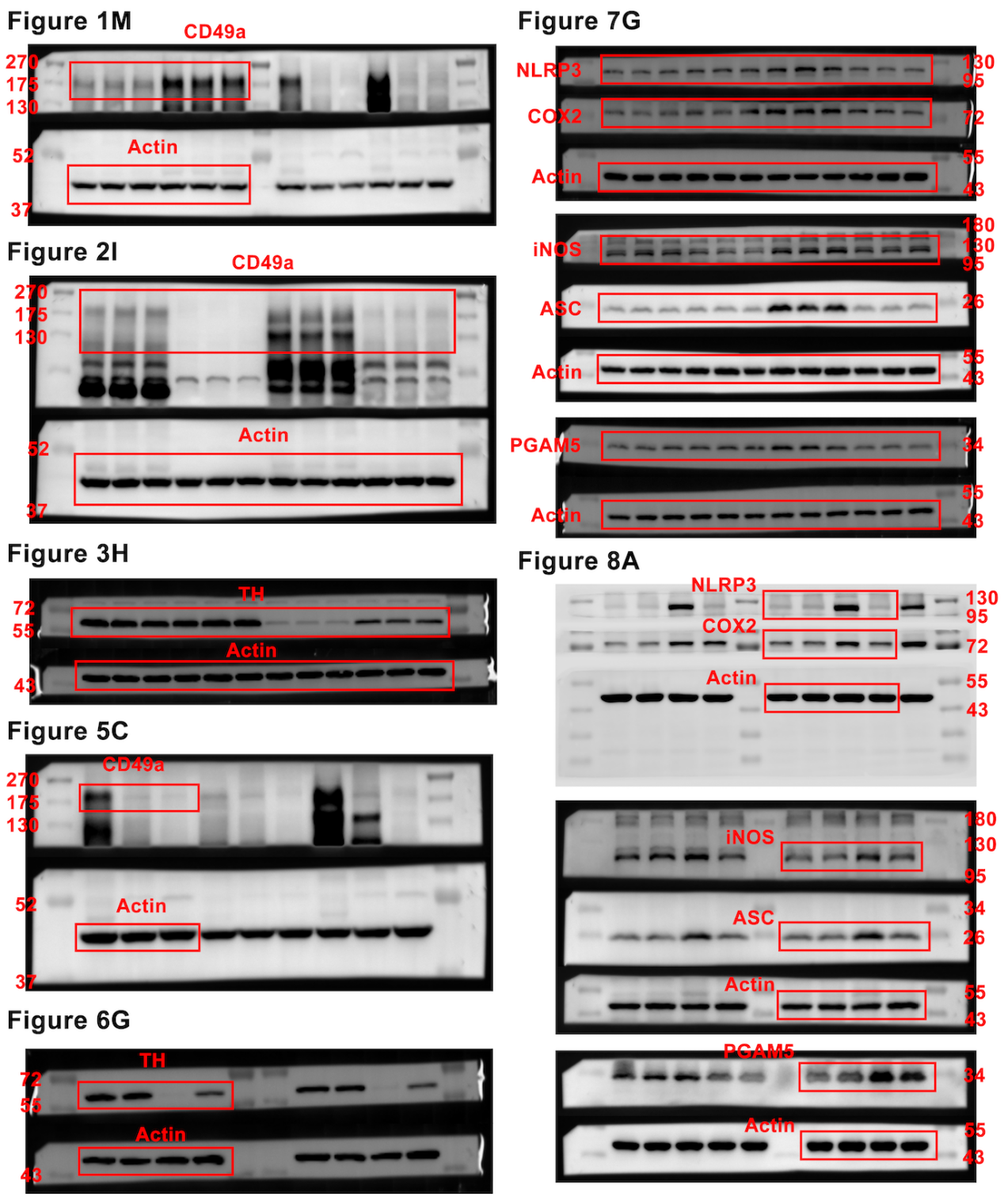


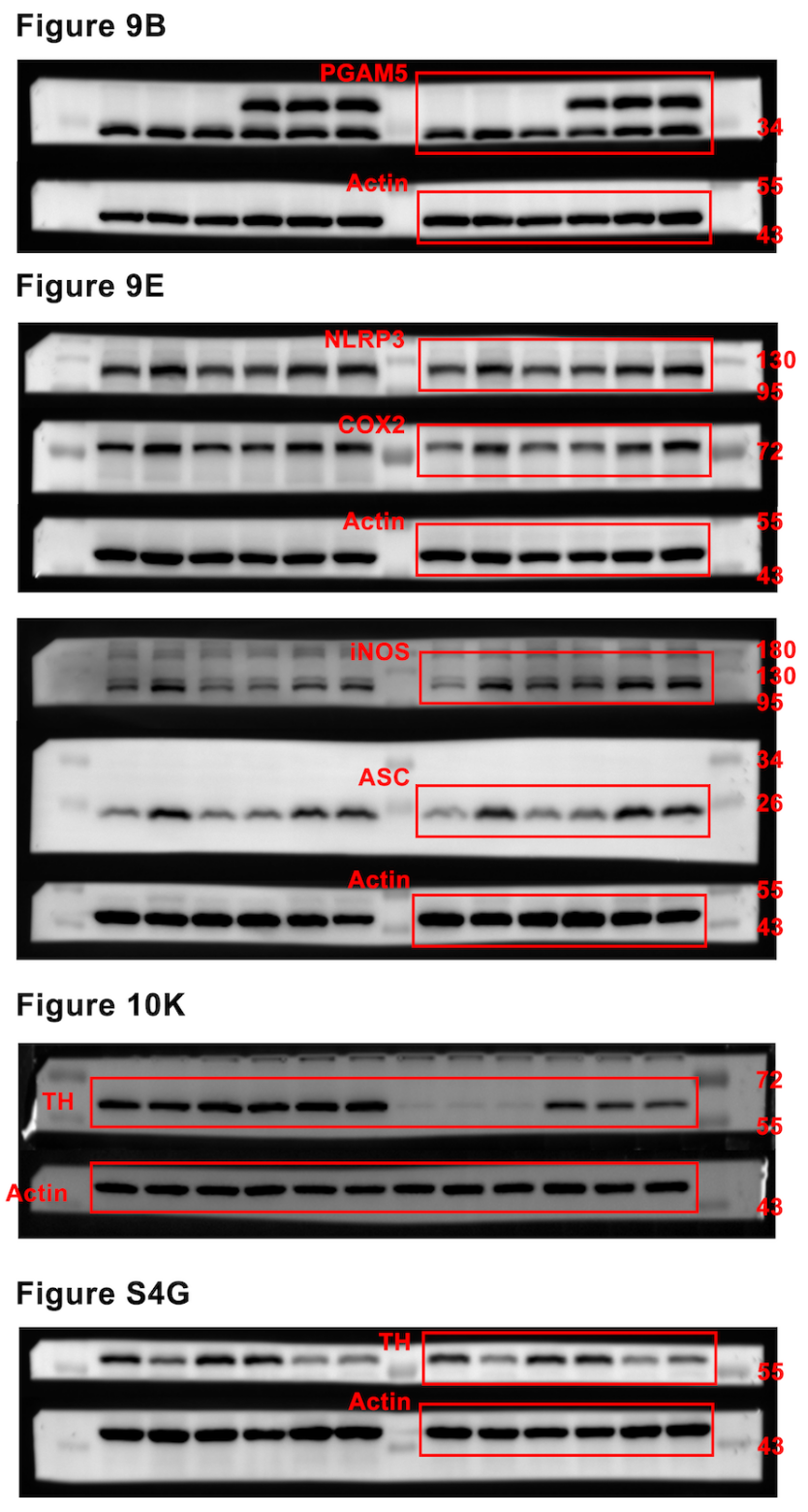

Supplement: Supplementary file 2 — Supporting File 2: advs73461‐sup‐0002‐DataFile.zip. [file ADVS-13-e15138-s001.zip › R3-Uncropped Blots.docx]
